# Research landscape and trends of lung cancer radiotherapy: A bibliometric analysis

**DOI:** 10.3389/fonc.2022.1066557

**Published:** 2022-11-10

**Authors:** Yanhao Liu, Shu Jiang, Yaru Lin, Haiming Yu, Lan Yu, Xiaotao Zhang

**Affiliations:** Department of Radiation Oncology, The Affiliated Qingdao Central Hospital of Qingdao University, Qingdao, China

**Keywords:** bibliometric analysis, lung cancer, radiotherapy, chemotherapy, chemoradiotherapy, SBRT, SAbR, immunotherapy

## Abstract

**Background:**

radiotherapy is one of the major treatments for lung cancer and has been a hot research area for years. This bibliometric analysis aims to present the research trends on lung cancer radiotherapy.

**Method:**

On August 31, 2022, the authors identified 9868 articles on lung cancer radiotherapy by the Web of Science (Science Citation Indexing Expanded database) and extracted their general information and the total number of citations. A bibliometric analysis was carried out to present the research landscape, demonstrate the research trends, and determine the most cited papers (top-papers) as well as top-journals on lung cancer radiotherapy. After that, the authors analyzed the recent research hotspots based on the latest publications in top-journals.

**Results:**

These 9868 papers were cited a total of 268,068 times. “Durvalumab after chemoradiotherapy in stage III non–small-cell lung cancer” published in 2017 by Antonia et al.was the most cited article (2110 citations). Among the journals, *New England Journal of Medicine* was most influential. Moreover, *J. Clin. Oncol.* and *Int. J. Radiat. Oncol. Biol. Phys.* was both influential and productive. Corresponding authors represented the USA (2610 articles) and China mainland (2060 articles) took part in most publications and articles with corresponding authors from Netherlands were most cited (46.12 citations per paper). Chemoradiotherapy was the hottest research area, and stereotactic body radiotherapy has become a research hotspot since 2006. Radiotherapy plus immunotherapy has been highly focused since 2019.

**Conclusions:**

This bibliometric analysis comprehensively and quantitatively presents the research trends and hotspots based on 9868 relevant articles, and further suggests future research directions. The researchers can benefit in selecting journals and in finding potential collaborators. This study can help researchers gain a comprehensive picture of the research landscape, historical development, and recent hotspots in lung cancer radiotherapy and can provide inspiration for future research.

## Introduction

Lung cancer is the leading cause of cancer-related death worldwide in both sexes (1). The two main pathologic subtypes of lung cancer are non-small-cell lung cancer (NSCLC, ~85% of cases) and small-cell lung cancer (SCLC, ~15% of cases) (2). Early lung cancer can be successfully treated but advanced disease is associated with poor prognosis as treatment options are limited. Radiotherapy is an effective treatment for lung cancer. In the 1990s, concurrent chemoradiotherapy was established as the standard of care for locally advanced unresectable lung cancer (3, 4), with stereotactic body radiotherapy (SBRT) used for early-stage NSCLC starting in the 2000s (5). Today, most patients with lung cancer receive radiotherapy as part of their therapeutic regimen. Thousands of articles on lung cancer radiotherapy have been published spanning research areas such as chemoradiotherapy, SBRT, neoadjuvant radiotherapy, and adjuvant radiotherapy. It is necessary but challenging for researchers to identify the most influential papers or to stay informed of research trends. Therefore, a comprehensive and quantified study is needed that systemically summarizes important advances, presents the current research hotspots, and suggests research directions.

As a method for sorting published articles and establishing the citation relationship (ie, bibliographic coupling) between them, a bibliometric analysis can aid researchers to become familiar with the state of a research area (6, 7). It is superior to review or meta-analysis in evaluating a whole academic discipline including thousands of publications (8). Therefore, a comprehensive bibliometric analysis of radiotherapy for lung cancer is needed. ​In the present study, we performed a bibliometric analysis based on articles published between 2000 and August 31, 2022 related to radiotherapy for lung cancer. The objectives of this study are: 1) describing the research status; 2) identifying the most influential articles (top-papers) and journals (top-journals); 3) evaluating the contribution of the countries, institutions, and authors; 4) demonstrating the research trends and latest research hotspots; 5) summarizing the important advances; and 6) suggesting future research direction.

## Methods

### Study selection

The Web of Science (Science Citation Indexing Expanded database) is frequently used for bibliometric analysis. This database includes more than 10,000 high-quality journals and comprehensive citation records (6). In addition, its document type labels of publications were reported to be more precise than other databases such as Scopus (9). Therefore, we chose Web of Science (Science Citation Indexing Expanded database) for the literature search.

We conducted a literature search on August 31, 2022 without restrictions on language. The time span was 2000–2022 and the article type was article. We designed the search strategy following some principles and performed multiple tests and modifications to identify as many relevant articles as possible while excluding irrelevant publications. The detailed search strategy and design principles were presented in **Supplementary Material S1**. We used Web of Science to extract and analyze the year of publication, journal, country/region, institution, total number of citations, and average number of citations per year. We then ranked the articles with the citation number to identify the 100 top-papers.

### Statistical analysis

Microsoft Office Excel 2019 software (Microsoft, Redmond, WA, USA) was used for descriptive statistical analysis and to produce tables. To demonstrate and visualize the research trends, the authors classified the articles by searching for research topics (and their synonyms) in titles and abstracts. Microsoft Visual Basic for Applications was used to perform a macro for data arrangement and batch retrieval. The “bibliometrix” package (v4.0.0) of R software (v4.2.1) was used for bibliometric analysis and data visualization. An online platform (https://bibliometric.com) was used to visualize international cooperation, and another online platform (https://www.citexs.com) was used to visualize the trends of keyword frequencies. The VOSviewer v1.6.17 software was used to construct a bibliographic coupling network based on the relationship between journal, country, co-authors, and keywords, and for network visualization and analysis. The authors established a thesaurus dictionary of keywords to merge the synonyms in the network visualization. The CiteSpace software (v6.1.R2) was used to identify keywords and references with the strongest citation bursts, to construct visualization maps of co-cited references and keywords, and to plot a dual-map overlay of journals.

The authors identified the journals that published the top-papers, and calculated their top-papers rates (TPR, the percentage of top-papers among all relevant papers in a journal). As the latest top-paper was published in 2019, the publication time span of the papers used to calculate TPR was limited to 2000–2019. Journals with a TPR >2% were considered the top-journals on lung cancer radiotherapy. Articles on lung cancer radiotherapy published in top-journals since 2020 were identified and analyzed to assess recent research hotspots.

## Results

**Figure 1:**
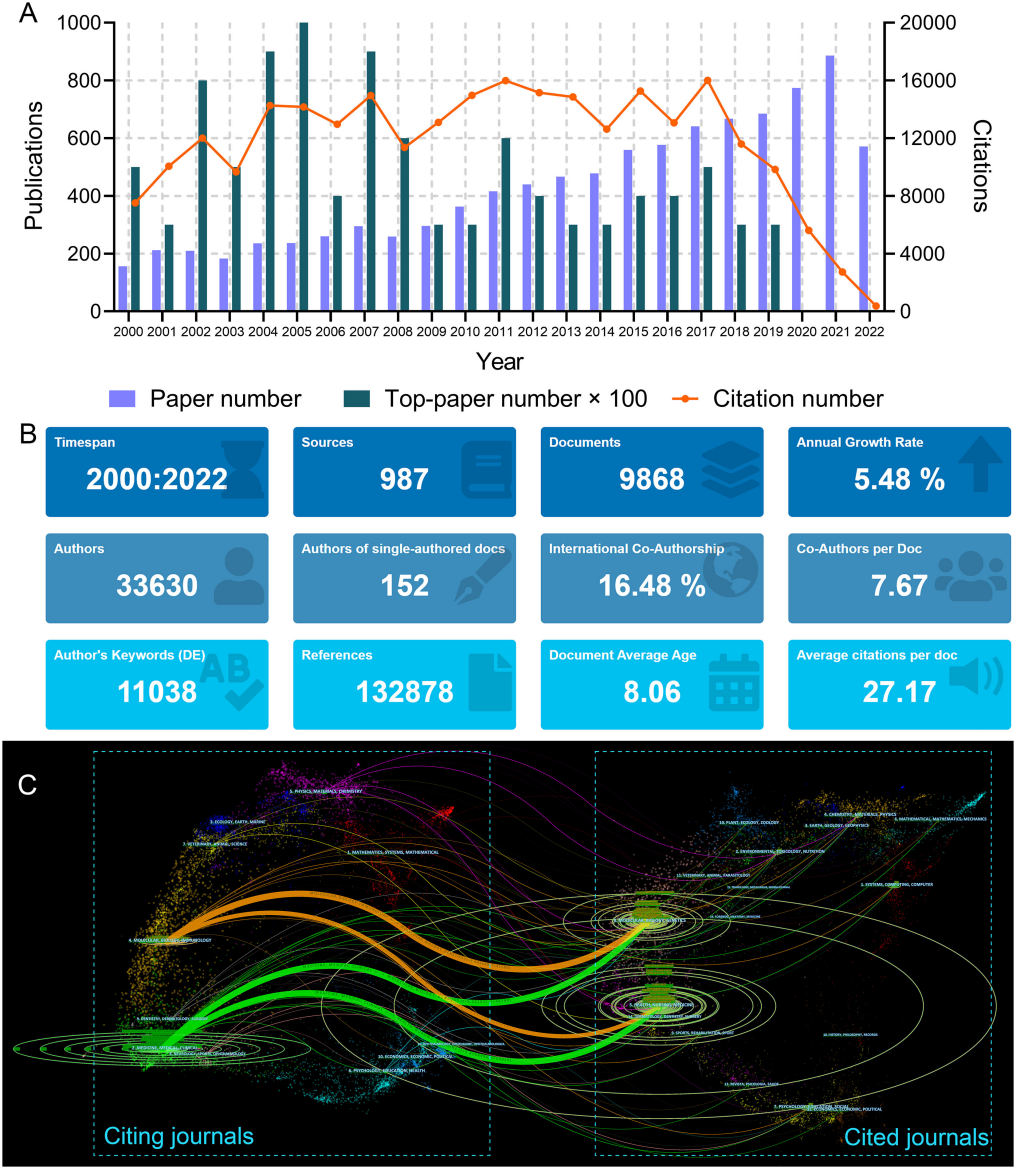
**(A)** Article number, top-paper number, and citation number from 2000 to 2022 of the articles on lung cancer radiotherapy. **(B)** General information of the 9868 articles on lung cancer radiotherapy. **(C)** The dual-map overlay of journal categories. The curves represent the citation relationship.

The search strategy returned 9868 articles (**Figure 1A**). The total number of citations for these papers was 268,068, and the median number of citations was 10. The historical direct citation network among the articles is shown in **Supplementary Figure S1**. A total of 132,878 references were cited by these articles (**Figure 1B**). The 50 references with the strongest citation bursts are listed in **Supplementary Figure S2**. The bibliographic coupling network of the most co-cited references is shown in **Supplementary Figure S3**.

The authors ranked the papers with the citation number and identified 100 top-papers (**Supplementary Table S1**). These articles were cited 49,464 times, which was 18.45% of the number of papers cited on lung cancer radiotherapy. Most of the top-papers (65 papers) were published between 2000 and 2010. “Durvalumab after chemoradiotherapy in stage III non–small-cell lung cancer” by Antonia et al., published in *The New England Journal of Medicine* (*N. Engl. J. Med.*) in 2017, had the highest average number of citations per year (436.55 times) and number of total citations (2110 times) (10). “Stereotactic body radiation therapy for inoperable early stage lung cancer” by Timmerman et al., published in *The Journal of the American Medical Association* (*JAMA; J. Am. Med. Assoc.*) in 2010, had the second highest number of total citations (1702 times) and sixth highest average number of citations per year (154.73 times) (11). “Durvalumab plus platinum-etoposide versus platinum-etoposide in first-line treatment of extensive-stage small-cell lung cancer (CASPIAN): a randomised, controlled, open-label, phase 3 trial” by Paz-Ares et al., published in *Lancet* in November 2019 (12), was the most recent publication among the top-papers. The 10 most cited articles were listed in **Table 1**.

**Table 1:** The 10 most cited papers in lung cancer radiotherapy from 2000 to 2022^a^.

Rank	Title	Corresponding Author	Journal	Year	Total citations	Average citations per year (rank)
1	Durvalumab after Chemoradiotherapy in Stage III Non-Small-Cell Lung Cancer	Antonia SJ	N. Engl. J. Med.	2017	2110	436.55 (1)
2	Stereotactic Body Radiation Therapy for Inoperable Early Stage Lung Cancer	Timmerman R	JAMA-J. Am. Med. Assoc.	2010	1805	144.4 (6)
3	Cisplatin-based adjuvant chemotherapy in patients with completely resected non-small-cell lung cancer	LeChevalier T	N. Engl. J. Med.	2004	1732	92.79 (13)
4	Overall Survival with Durvalumab after Chemoradiotherapy in Stage III NSCLC	Antonia SJ	N. Engl. J. Med.	2018	1358	362.13 (2)
5	Standard-dose versus high-dose conformal radiotherapy with concurrent and consolidation carboplatin plus paclitaxel with or without cetuximab for patients with stage IIIA or IIIB non-small-cell lung cancer (RTOG 0617): a randomised, two-by-two factorial phase 3 study	Bradley JD	Lancet Oncol.	2015	1240	163.52 (4)
6	Adjuvant vinorelbine plus cisplatin versus observation in patients with completely resected stage IB-IIIA non-small-cell lung cancer (Adjuvant Navelbine International Trialist Association [ANITA]): a randomised controlled trial	Douillard JY	Lancet Oncol.	2006	1153	72.06 (18)
7	Excessive toxicity when treating central tumors in a phase II study of stereotactic body radiation therapy for medically inoperable early-stage lung cancer	Timmerman R	J. Clin. Oncol.	2006	1074	67.48 (23)
8	Radiotherapy plus chemotherapy with or without surgical resection for stage III non-small-cell lung cancer: a phase III randomised controlled trial	Albain KS	Lancet	2009	993	75.9 (15)
9	Akt/protein kinase B is constitutively active in non-small cell lung cancer cells and promotes cellular survival and resistance to chemotherapy and radiation	Dennis PA	Cancer Res.	2001	812	38.06 (62)
10	Lung Cancer: Epidemiology, Etiology, and Prevention	DelaCruz CS	Clin. Chest Med.	2011	802	74.6 (16)

### Journals

**Figure 2:**
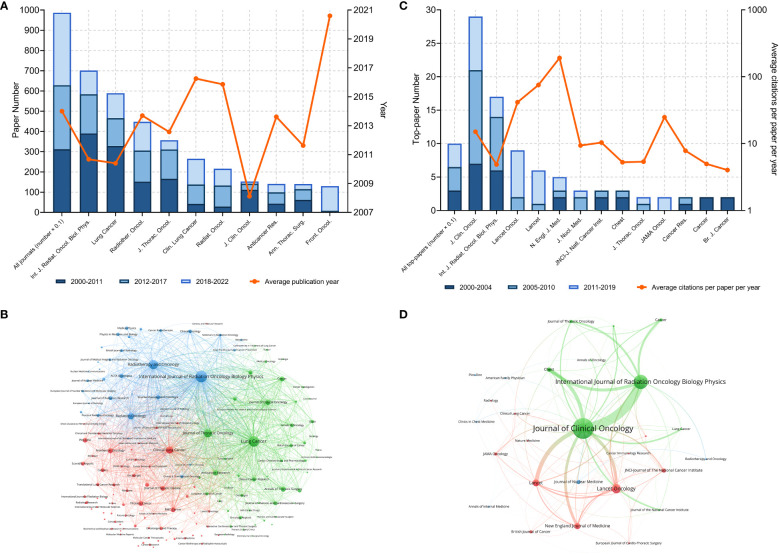
**(A)** Article numbers and average publication year of the articles of the top-10 productive journals. **(B)** Bibliographic coupling of journals with at least ten papers related to lung cancer radiotherapy. **(C)** Top-paper number and average citation per paper per year of the journals with at least two top-papers. **(D)** Bibliographic coupling of journals with top-papers related to lung cancer radiotherapy. In the bibliographic coupling maps, the circle size represents the number of papers. The breadth of the curves represents the connection strength. The journals in the same color are of similar research areas.

A total of 987 journals published articles on lung cancer radiotherapy. A dual-map overlay showed the academic discipline distribution and the citation relationship of these journals (**Figure 1C**). This map revealed that articles on molecular/biology/immunology or medicine/medical/clinical mainly cited articles on molecular/biology/genetics or health/nursing/medicine. Among the journals, *International Journal of Radiation Oncology Biology Physics* (*Int. J. Radiat. Oncol. Biol. Phys.*) (701 articles), *Lung Cancer* (589 papers), and *Radiotherapy and Oncology* (448 papers) were the top three journals with the most articles (**Figure 2A**). Among the 10 most productive journals, *Journal of Clinical Oncology (J. Clin. Oncol.)* had the highest average number of citations per paper (159.12), the average number of citations per article per year (15.08) and impact factor (50.72), which indicated that it was both productive and influential (**Table 2**). *Int. J. Radiat. Oncol. Biol. Phys.* Had the highest total citation (39340) and local citation (30064). Papers in *Frontiers in Oncology* had an average publication year of 2020.6 and most of them were published after 2018, which indicated that *Frontiers in Oncology* is a rising journal in this area. Moreover, the top 10 journals with highest citation per paper per year and at least five articles were identified (**Table 3**). *New England Journal of Medicine (N. Engl. J. Med.)* had the highest citation per paper per year (191.09). In particular, only *J. Clin. Oncol.* was both highly productive and influential in this area. The bibliographic coupling map of journals related to lung cancer radiotherapy was conducted (**Figure 2B** **)**.

**Table 2:** The top 10 productive journals in lung cancer radiotherapy from 2000 to 2022.

Journals	Paper number	Total citation	Citation per paper	Citation per paper per year^a^	Average publication year	Local citation^a^	IF (2021)
Int. J. Radiat. Oncol. Biol. Phys.	701	39340	56.12	4.89	2010.67	30064	8.01
Lung Cancer	589	15551	26.40	2.75	2010.40	2142	6.08
Radiother. Oncol.	448	15439	34.46	3.95	2013.69	10428	6.90
J. Thorac. Oncol.	357	14390	40.31	5.38	2012.57	9162	20.12
Clin. Lung Cancer	265	3615	13.64	2.52	2016.26	2387	4.84
Radiat. Oncol.	216	3876	17.94	2.64	2015.86	2148	4.31
J. Clin. Oncol.	153	24346	159.12	15.08	2008.12	23597	50.72
Anticancer Res.	141	1218	8.64	1.25	2013.61	1197	2.44
Ann. Thorac. Surg.	140	5127	36.62	3.29	2011.63	5225	5.10
Front. Oncol.	130	355	2.73	1.29	2020.6	775	5.74

a Citation number in the current dataset (papers in lung cancer radiotherapy from 2000 to 2022).

**Table 3:** The top 10 journals with highest citation per paper per year in lung cancer radiotherapy from 2000 to 2022^a^.

Journals	Paper number	Top-Paper number	Top-Paper rate^b^	Total citation	Citation per paper	Citation per paper per year	Local citation^c^	IF (2021)
*N. Engl. J. Med.*	5	5	100.00%	6269	1253.80	191.09	8423	176.08
*Lancet*	7	6	85.71%	3635	519.29	75.47	3070	202.73
*Lancet Oncol.*	29	9	31.03%	7461	257.28	41.57	4248	54.43
*JAMA Oncol.*	24	2	8.33%	1846	76.92	24.80	926	33.01
*J. Clin. Oncol.*	153	29	18.95%	24346	159.12	15.08	23597	50.72
*JNCI*	14	3	21.43%	2034	145.29	10.38	1815	11.82
*J. Nucl. Med.*	39	3	7.69%	3005	77.05	9.39	1734	11.08
*Clin. Chest Med.*	9	1	11.11%	933	103.67	9.36	171	4.97
*Radiology*	14	1	7.14%	1199	85.64	8.49	1952	29.15
*Cancer Res.*	29	2	6.90%	2993	103.21	7.84	4996	13.31

a Only journals with at least five papers related to lung cancer radiotherapy were included.

b Percentage of top- papers among all papers in a journal. The time span was from 2010 to 2019 (the publication year of the lastest top- paper).

c Citation number in the current dataset (papers in lung cancer radiotherapy from 2000 to 2022).

The 100 top-papers were published in 28 journals. Among them, 13 journals published at least 2 top-papers (**Figure 2C**). The bibliographic coupling map of journals with top-papers was conducted (**Figure 2D** **)**. *J. Clin. Oncol.* published most top-papers (29 papers), followed by *Int. J. Radiat. Oncol. Biol. Phys.* (17 papers) and *Lancet Oncology* (9 papers). The TPRs of the 28 journals were calculated (**Supplementary Table S2**). A total of 20 journals with a TPR of at least 2% were therefore considered as top-journals in this area. Papers in this area published in top-journals are highly likely to be influential. Notably, the TPR of *N. Engl. J. Med.* was 100%. Since 2020, 110 articles have been published in the top-journals (**Supplementary Table S3**).

### Countries/Regions

**Figure 3:**
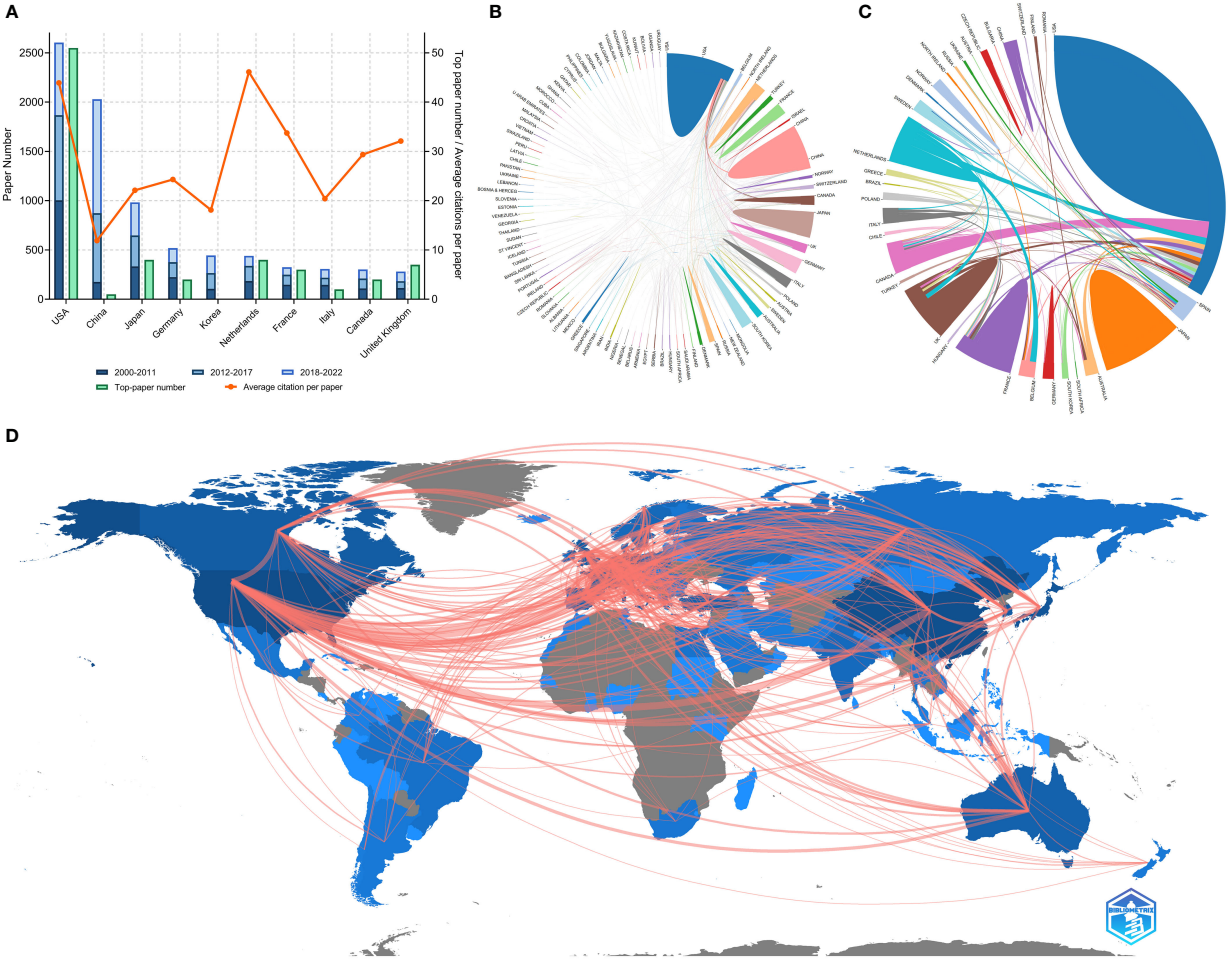
**(A)** Paper number, top-paper number, and average citations per paper of corresponding authors’ countries. **(B)** Network mapping of international collaboration base on 9868 papers related to lung cancer radiotherapy. **(C)** Network mapping of international collaboration base on 100 top-papers related to lung cancer radiotherapy. **(D)** Visualization world map of publications and collaboration relationship.

**Figure 4:**
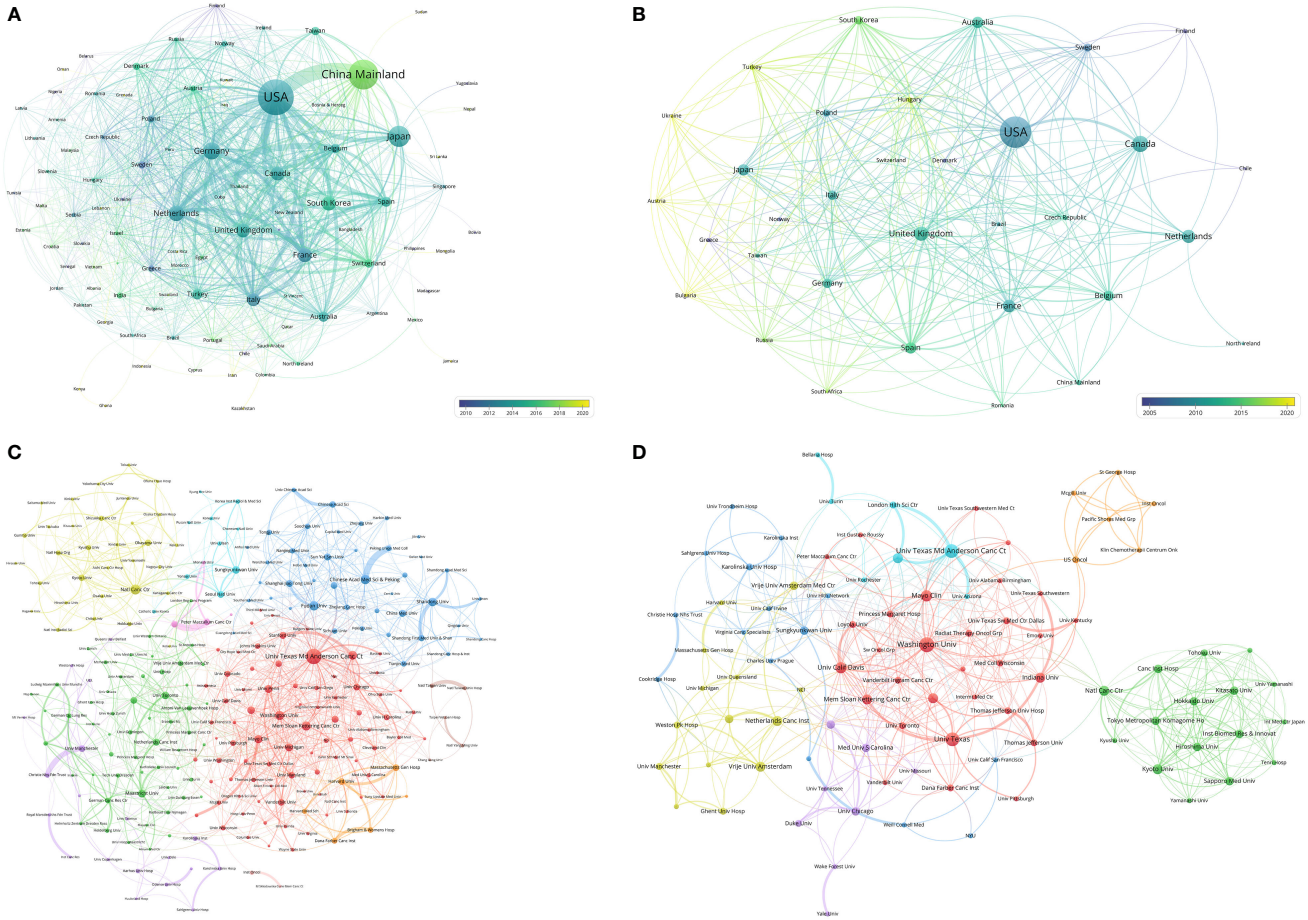
**(A)** Network visualization of countries with papers related to lung cancer radiotherapy. **(B)** Network visualization of countries with top-papers related to lung cancer radiotherapy. **(C)** Network visualization of institutions with at least 20 articles related to lung cancer radiotherapy. **(D)** Network visualization of institutions with at least 2 top-papers related to lung cancer radiotherapy. The circle size represents the number of papers. The breadth of the curves represents the connection strength. The institutions in the same color have stronger collaboration with each other.

Researchers from 100 countries/regions contributed to the 9868 articles. However, the corresponding authors of these articles only from 71 of the countries/regions and only 48 countries/regions contributed to at least 10 articles. The corresponding authors from the United States contributed the most publications (2610 papers), followed by the corresponding authors from China Mainland (2060 papers) and Japan (989 papers) (**Table 4** and **Figure 3A**). Papers by corresponding authors from the United States were cited as high as 114,594 times. The citation per paper of Netherlands was the highest (46.12). Authors of most articles were from single countries. International collaboration was more common in North American or European countries than in Asian countries. A chordal graph and a network world map were conducted to visualize the international collaboration in this area (**Figures 3B, D**). A network visualization map showed the co-author relationship of the countries/regions (**Figure 4A**). Most articles by low-income countries/regions were published more recently than those of other countries.

**Table 4:** The top 10 productive countries of corresponding authors of papers in lung cancer radiotherapy from 2000 to 2022.

Countries	Paper number	Percentage (N/2941)	Multiple-country paper rate^b^	Total citation	Citation per paper	Top-paper number^a^	Top-paper rate	Multiple-country top-paper rate
USA	2610	26.45%	17.43%	114594	43.91	51	1.95%	39.22%
China Mainland	2060	20.88%	8.93%	24484	11.89	1	0.05%	0.00%
Japan	989	10.02%	3.84%	21871	22.11	8	0.81%	12.50%
Germany	525	5.32%	25.90%	12758	24.30	4	0.76%	0.00%
Korea	447	4.53%	7.38%	8096	18.11	0	0	/
Netherlands	443	4.49%	32.96%	20433	46.12	8	1.81%	50.00%
France	326	3.30%	12.58%	10997	33.73	6	1.84%	50.00%
Italy	310	3.14%	16.45%	6327	20.41	2	0.65%	50.00%
Canada	302	3.06%	27.15%	8868	29.36	4	1.32%	100.00%
United Kingdom	282	2.86%	19.50%	9053	32.10	7	2.48%	42.86%

a Besides the countries mentioned above, corresponding authors from other seven countries contributed nine top-papers.

b Percentage of multiple-country top-papers among all papers of a country.

The top-papers were published by authors from 33 countries/regions and corresponding authors from 16 countries/regions. The corresponding auhors from the United States published more than a half of the top-papers (51 papers). International collaboration was common in the-top papers (**Figure 3C**). A network visualization map showed the co-author relationship of the countries/regions with the top-papers (**Figure 4B**).

### Institutions

The authors of the 9868 articles represented 8109 institutions. The University of Texas MD Anderson Cancer Center contributed most articles (971 papers) among institutions (**Table 5**). Six of the 10 most productive institutions were in the United States and the two were in China Mainland. A collaboration network and a cluster analysis of the co-author relationship of institutions were conducted (**Figure 4C**). Most institutions preferred domestic collaboration over international collaboration. International collaboration was common between the institutions with the strongest research strength in their countries.

**Table 5:** The top 10 institutions with the most papers or top-papers on lung cancer radiotherapy from 2000 to 2022.

Institutions	Country/Region	Paper number^a^	Percentage (N/9868, %)	Top-paper number	Top-paper rate	Top-paper number rank
Univ Texas MD Anderson Canc Ctr	USA	971	9.84%	18	1.85%	1
Fudan Univ	China Mainland	397	4.02%	0	0.00%	/
Mem Sloan Kettering Canc Ctr	USA	376	3.81%	9	2.39%	8
Sungkyunkwan Univ	Korea	352	3.57%	4	1.14%	43
Netherlands Canc Inst	Netherlands	317	3.21%	11	3.47%	5
Shandong Univ	China Mainland	315	3.19%	0	0.00%	/
Duke Univ	USA	301	3.05%	5	1.66%	22
Univ Texas	USA	297	3.01%	12	4.04%	3
Washington Univ	USA	287	2.91%	15	5.23%	2
Univ Michigan	USA	273	2.77%	5	1.83%	22
Indiana Univ	USA	73	0.74%	11	15.07%	5
Vrije Univ Amsterdam	Netherlands	183	1.85%	11	6.01%	5
Natl Yang Ming Univ	Taiwan	62	0.63%	9	14.52%	8
Univ Colorado	USA	140	1.42%	9	6.43%	8

a All papers were included, without limitation of corresponding author’s institutions.

A total of 451 institutions contributed to top-papers. The three leading productive institutions of the top-papers were the University of Texas MD Anderson Cancer Center (18 papers), Washington University (15 papers), and University of Texas (12 papers). In particular, Indiana University (15.07%) and National Yang-Ming University (14.52%) had high TPRs. Although some institutions in China published many articles, their top-paper number was low. A collaboration network and cluster analysis of the co-author relationship of institutions with top-papers was conducted (**Figure 4D**). In contrast to the clusters in **Figure 4C**, the cluster boundaries of the institutions with top-papers were obscure. Collaboration between institutions with top-papers was common and less restricted by geographical parameters. However, the collaboration between Japanese institutions and others remained rare.

### Authors

**Figure 5:**
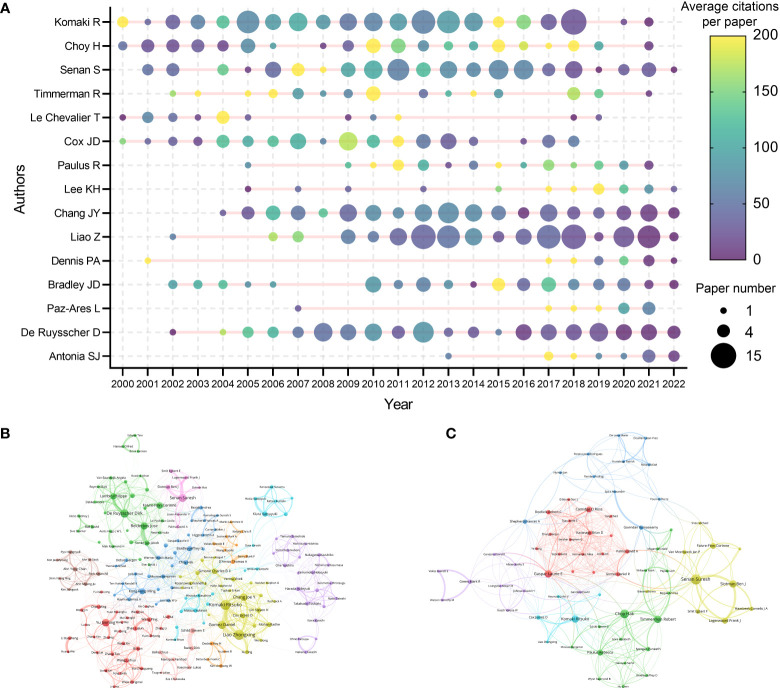
**(A)** The top-15 cited authors’ production and citation over time. The node size represents the paper number and the color represents the average citations per paper. **(B)** Network visualization of authors with at least 20 papers related to lung cancer radiotherapy. **(C)** Network visualization of authors with at least 2 top-papers related to lung cancer radiotherapy. The circle size represents the number of papers. The breadth of the curves represents the connection strength. The authors in the same color have stronger collaboration with each other.

A total of 33630 authors contributed to the 9868 articles. Komaki R was the author with the highest citations (10873 citations) and H-index (13) (**Table 6**). Choy H was the most local cited author (2175 local citations). Besides, some researchers such as Senan S, Paulus R, Chang JY, and Nagata Y were also highly impactful in this area. The production and citation number over time of the 15 most cited authors was visualized (**Figure 5A** **)**. Some of the most cited authors, such as Komaki R and Choy H, consistently produced articles in the last two decades. Moreover, the most impactful articles by some other authors, such as Lee KH and Paz-Ares L, have been published in recent years. Notably, although only published 11 papers related to lung cancer radiotherapy, Dennis PA was the 10th cited author (4677 citations). A collaboration network map and clustering analysis showed the co-authors relationship (**Figure 5B**). Researchers in Asia preferred to collaborate with researchers in their own countries rather than with foreign researchers.

**Table 6:** The most impactful authors related to lung cancer radiotherapy from 2000 to 2022^a^.

Name	Paper number	Total citation	Name	Local citation	Name	H-index	Name	Top-paper number
Komaki R	136	10873	Choy H	2175	Komaki R	55	Choy H	7
Choy H	58	7769	Senan S	1734	Senan S	44	Senan S	7
Senan S	102	7418	Paulus R	1625	Chang JY	39	Nagata Y	5
Le Chevalier T	17	6175	Komaki R	1566	Liao Z	38	Slotman BJ	5
Cox JD	59	5884	Bradley JD	1219	Cox JD	36	Komaki R	4
Paulus R	24	5877	Timmerman R	1168	De Ruysscher D	36	Lagerwaard FJ	4
Lee KH	15	4874	De Ruysscher D	1013	Lambin P	33	Le Chevalier T	4
Chang JY	94	4811	Green MR	923	Choy H	31	Paulus R	4
Liao Z	116	4787	Bezjak A	838	Bradley JD	29	Hayakawa K	4
Dennis PA	11	4677	Hu C	837	Lagerwaard FJ	29	Hiraoka M	4

a As many authors had duplicate English names (e.g. Wang Y), the most productive authors were not presented.

Analysis of corresponding authors highlighted the main contributors to the studies. A total of 5694 corresponding authors were recognized. As corresponding author, Yu JM contributed to most papers (43 papers) (**Table 7**). Timmerman R was the most cited corresponding author with 8 articles (3767 citations). In particular, Antonia SJ was the corresponding author of only two articles, but these articles were cited as high as 3468 times. Moreover, Chang JY, the corresponding author of 33 articles (2104 citations), was both highly productive and influential.

**Table 7:** The top 10 productive and cited corresponding authors in lung cancer radiotherapy from 2000 to 2022.

**Most productive corresponding author**	**Paper number**	**Total citation**	**Average citations per paper**	**Top-paper number**	**Most cited corresponding author**	**Paper number**	**Total citation**	**Average citations per paper**	**Top-paper number**
Yu JM	43	695	16.16	0	Timmerman R	8	3767	470.88	3
Rades D	39	464	11.90	0	Antonia SJ	2	3468	1734.00	2
Liao ZX	36	1902	52.83	1	Bradley JD	18	2887	160.39	1
Kong FM	35	1696	48.46	1	Chang JY	33	2104	63.76	0
Lu B	34	1380	40.59	0	Lagerwaard FJ	16	2061	128.81	3
Chang JY	33	2104	63.76	0	Onishi H	8	2029	253.63	3
Li BS	29	533	18.38	0	Liao ZX	36	1902	52.83	1
DeRuysscher D	27	1765	65.37	0	LeChevalier T	3	1797	599.00	1
Wang LH	26	548	21.08	0	DeRuysscher D	27	1765	65.37	0
Jeremic B	23	249	10.83	0	Kong FM	35	1696	48.46	1

A total of 1246 authors contributed to the 100 top-papers. Choy H and Senan S was the most productive authors of the top-papers (7 papers each), followed by Nagata Y and Slotman BJ (5 papers each). A collaboration network and clustering analysis showed the co-author relationship of the top-papers (**Figure 5C**). International collaboration between these authors was common. A total of 12 researchers were corresponding authors of at least two top-papers (**Supplementary Table S4**). Timmerman R, Onishi H, and Lagerwaard FJ was the most productive corresponding authors of top-papers (3 top-papers each).

### Keywords

**Figure 6:**
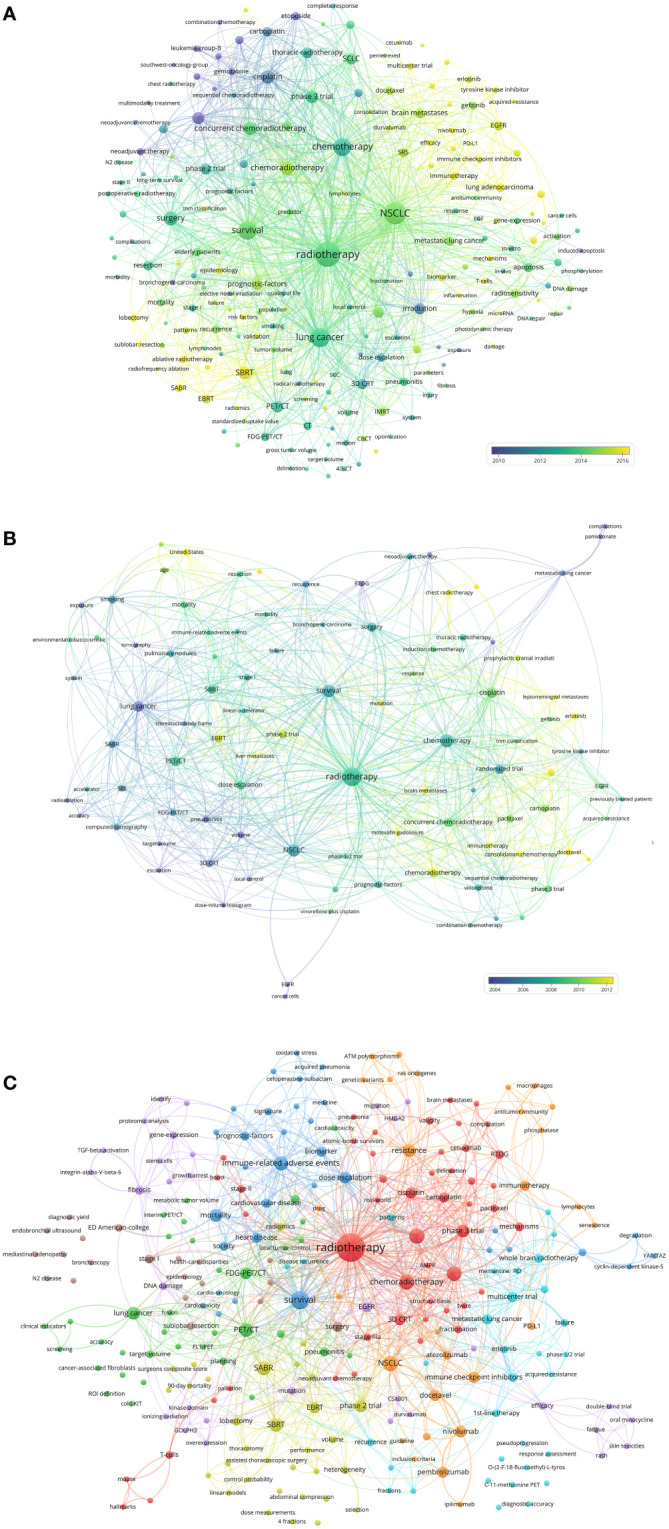
**(A)** Network visualization of keywords that occurred at least 50 times in the 9868 articles. **(B)** Network visualization of keywords that occurred at least twice in the top-papers. **(C)** Network visualization of keywords in articles published in top-journals between 2020 and 2022. The circle size represents the number of papers. The breadth of the curves represents the connection strength.

The authors analyzed the hot keywords in multiple dimensions based on the author-selected keywords and keyword-plus identified by Web of Science. The frequency rank variation of keyword occurrence in lung cancer radiotherapy between 2000 and 2022 was shown in **Supplementary Figure S4**. “immunotherapy”, “SBRT”, and “brain metastases” are considered as emerging hot keywords. The authors identified the top 50 keywords with the strongest citation bursts (**Supplementary Figure S5** **)**. In recent years, “immunotherapy” and “SBRT” have become hotspots. The keyword co-occurrence network of the 9868 articles was conducted (**Figure 6A**). The top-keywords included “chemoradiotherapy”, “clinical trial”, “prognostic-factors”, “cisplatin”, and “PET/CT”. The emerging keywords included “immune-related adverse events”, “SBRT”, “immune checkpoint inhibitors”, “EMT”, “autophagy”, “proton therapy”, and “oligometastasis”.

The keyword co-occurrence network of the top-papers was conducted (**Figure 6B**). The newly utilized keywords included “randomized trial”, “sequential chemoradiotherapy”, “leptomeningeal metastases”, “hyperfractionated radiotherapy”, and “liver metastases”. The keyword co-occurrence network of the 110 recently published articles in top-journals was conducted (**Figure 6C**). The emerging hot keywords included “acquired-resistance”, “AMPK”, “atezolizumab”, “ATM polymorphisms”, “C-11-methionine PET”, “cardiac toxicity”, “N2 disease”, and “consolidation chemotherapy”.

### Research trends

**Figure 7:**
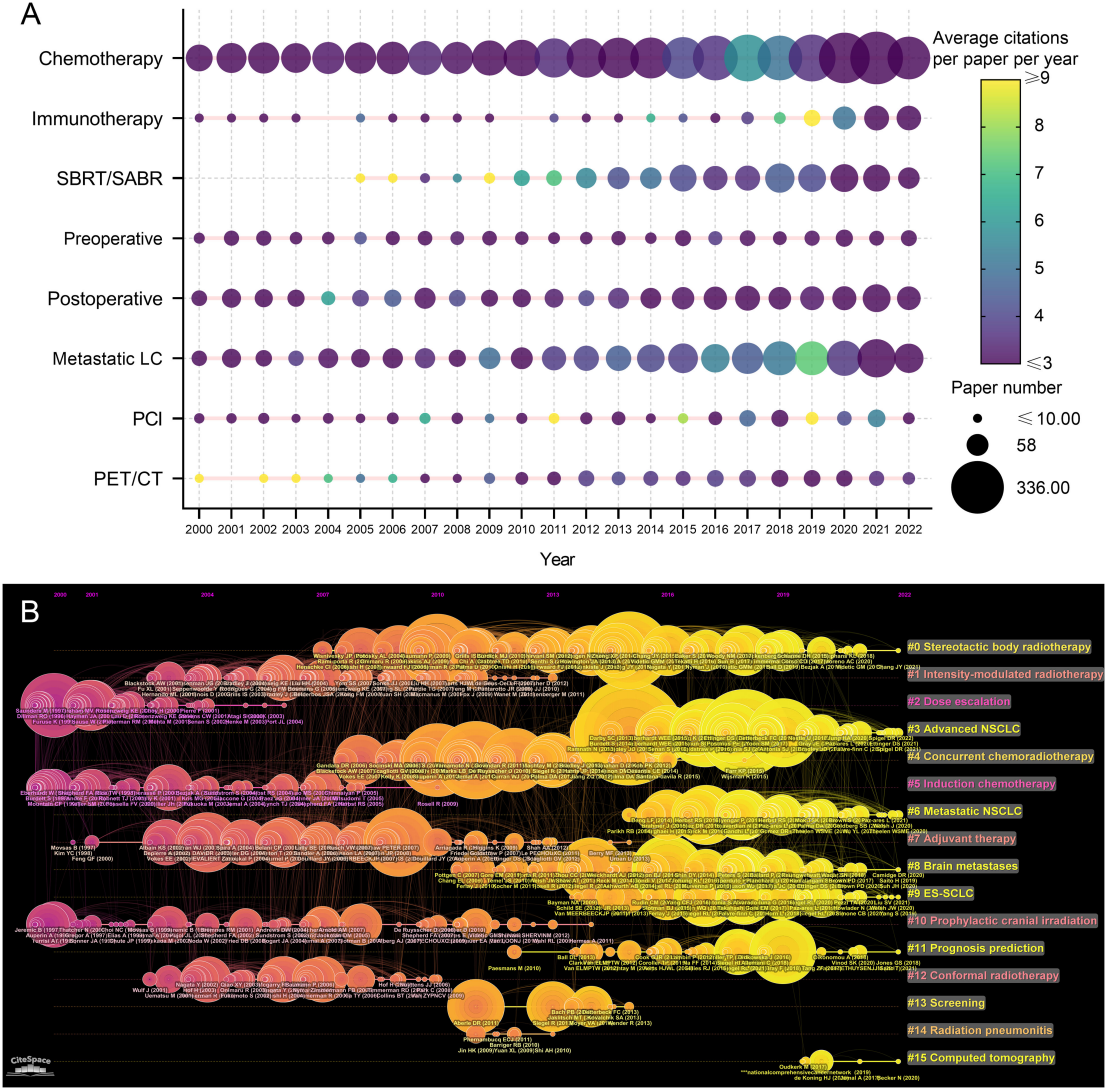
**(A)** Publication number and citations per paper per year of different research area related to lung cancer radiotherapy. The node size represents the paper number and the color represents the average citations per paper. **(B)** The timeline view for co-cited references related to lung cancer radiotherapy between 2000 and 2022. The node size represents the citation number of the reference. The curves between the nodes indicated co-citation relationships. Yellow nodes represent new papers and red nodes represent old ones.

The related research topics of the 9868 articles were identified based on titles and abstracts. The research topic variation between 2000 and 2022 were analyzed and visualized (**Figure 7A**). “Chemotherapy” was always the most popular topic in articles related to lung cancer radiotherapy, followed by “metastatic lung cancer” and “postoperative radiotherapy”. The number of articles on these topics has gradually increased over the past two decades. The number of articles related to immunotherapy has increased rapidly in recent years, and papers published in 2018 and 2019 were impactful. The influential pioneer articles in SBRT/SABR were published prior to 2010, and the paper number has increased significantly since then.

A timeline view of the co-cited reference variation was conducted (**Figure 7B**). The references were classified into 16 clusters. The clusters with large yellow nodes, which represented many newly published articles, were recent research hotspots. The recent popular topics included “SBRT”, “advanced NSCLC”, “metastatic NSCLC”, and “extensive-stage SCLC (E-SCLC)”.

## Discussion

Radiotherapy is a noninvasive therapeutic modality that has been used for the local treatment of lung cancer since the early 20th century. Early radiotherapy techniques were not conformal and resulted in severe complications. In the 1990s, three-dimensional conformal radiotherapy (3D-CRT) and intensity-modulated radiation therapy (IMRT) were applied to treat carcinomas (14, 15). With higher radiation doses delivered to tumors and less toxicity, 3D-CRT and IMRT constituted major advances in the radiotherapy-based treatment of lung cancer. In the 2000s, SBRT—a highly accurate technology—was successfully used to treat early-stage NSCLC (5). In recent years, the combination of radiotherapy with ICIs has further improved the prognosis of patients.

### Chemoradiotherapy for NSCLC

With improvements in the efficacy and safety of radiotherapy, radical radiotherapy became feasible. However, as a local treatment, radiotherapy alone may not prolong overall survival (OS). A randomized trial conducted in 1990 showed that induction chemotherapy plus radiotherapy was superior to radiotherapy alone in stage III NSCLC (16). Subsequent trials explored the optimal chemotherapeutic agents and doses, radiotherapy doses and fractions, and chemoradiotherapy order (17, 18). A randomized phase 3 trial conducted in 1999 showed that concurrent chemoradiotherapy (CCRT) significantly improved response and OS rates compared to sequential treatment in selected patients with unresectable stage III NSCLC (19), which was confirmed by later trials (20, 21). Since then, platinum-based CCRT has been the standard of care for unresectable stage III NSCLC.

Several studies have aimed to explore whether CCRT plus induction chemotherapy, consolidation chemotherapy, or tyrosine kinase inhibitors (TKIs) could further improve the prognosis of this patient population, but the results were negative (22–24). The results of a randomized phase 3 trial (RTOG 0617) showed that high-dose (74 Gy) radiotherapy with concurrent chemotherapy was not superior to standard CCRT (60 Gy) for stage III NSCLC patients; moreover, the addition of cetuximab provided no additional benefit (25, 26). In 2021, a phase 2 randomized trial (NRG-LU001) evaluated the efficacy of metformin plus CCRT for stage III NSCLC. However, the addition of metformin did not improve the prognosis (27).

Proton radiotherapy had some physical superiority to photon radiotherapy and was first used for the treatment of lung cancer in the 2000s (28). In 2011, a retrospective study compared proton-based CCRT with photon-based CCRT for locally advanced NSCLC. This study suggested that proton-based CCRT could deliver higher dose to tumor while resulting in lower toxicity (29). A phase 2 trial demonstrated proton-based CCRT had encouraging efficacy and safety for stage III NSCLC (30). In 2017, a propensity matched analysis based on National Cancer Database suggested that proton radiotherapy result in longer OS than photon radiotherapy for patients with stage II or III NSCLC (31). A randomized trial compared passive scattering proton therapy plus concurrent chemotherapy with photon-based CCRT for inoperable NSCLC, however, the results showed that the two approaches resulted in similar incidences of grade 3 radiation pneumonitis and local failure (32). Hypofractional radiotherapy might overcome the irradiation resistance of cancer, but hypofractional photon therapy resulted in prohibitive toxicities (33). Recently, some studies evaluated hypofractional proton therapy plus chemotherapy for stage III NSCLC. The efficacy was promising and the toxicity was acceptable, however, late serious adverse events occurred in some patients (33, 34).

Chemoradiotherapy is the most active lung cancer research area based on the number of published papers. CCRT has been the standard treatment for stage III NSCLC for years. In recent years, the optimization of CCRT is highly focused. Proton therapy can deliver a higher dose to the tumor than photon radiotherapy, but more clinical evidence is needed to further clarify the efficacy and safety, and determine the optimal dose fraction. Immune checkpoint inhibitors (ICIs) plus CCRT achieves encouraging efficacy (10), further studies are needed demonstrate the optimal timing and strategy of combining ICIs with CCRT.

### SBRT/SABR for early-stage NSCLC

SBRT, also known as stereotactic ablative radiotherapy (SABR), is used for the treatment of early-stage NSCLC. The first clinical trial of SBRT for early-stage NSCLC, which was reported in 2005, demonstrated excellent efficacy and safety (5); and a phase 2 trial conducted in 2010 showed that SBRT with a radiation dose of 54 Gy delivered in 3 fractions for early-stage NSCLC yielded a 3-year local control rate of 97.6%, 3-year OS rate of 55.8%, and grade 3/4 toxicity rate of 16.3% (11). This article was cited 1805 times and firstly established the standard of SBRT in treating inoperable early-stage NSCLC. A randomized phase 3 trial (TROG 09.02 CHISEL) reported that SABR resulted in superior local disease control without increased toxicity compared to standard radiotherapy in patients with peripheral stage I NSCLC (35). The most common recurrent pattern of SBRT for early-stage NSCLC was distant recurrences (36). There were two important questions concerning SBRT for early-stage NSCLC: 1) patient selection and dose limitation in the treatment of central lesions; and 2) the noninferiority of SBRT to surgery for operable disease (7).

SBRT (60–66 Gy in three fractions) has shown excessive toxicity in the treatment of early-stage NSCLC near the central airway (37); therefore, the dose and number of fractions must be optimized to improve safety. Haasbeek et al. (2011) used SBRT with a total dose of 60 Gy in eight fractions to treat 63 patients with central early-stage NSCLC, with 4 patients experiencing grade 3 chest wall pain or dyspnea (38). Chang et al. (2008, 2014) found that early-stage NSCLC patients who received SBRT (50 Gy in four fractions) had clinical outcomes similar to patients with peripheral NSCLC when normal tissue constraints were respected (39, 40). Single-fraction SBRT appears feasible for peripheral tumors: in a randomized phase 2 trial (RTOG 0915), SBRT with a total dose of 34 Gy in one fraction or 48 Gy in four fractions had comparable efficacy and safety for stage I peripheral NSCLC (41).

The noninferiority of SBRT to surgery for operable early-stage NSCLC was a research hotspot. Two retrospective studies compared SBRT and surgery for early-stage NSCLC, but reported opposite results (42, 43). A pooled analysis of two randomized phase 3 trials (STARS and ROSEL) indicated that SBRT was as effective and safe as surgery for early-stage NSCLC (44); however, the studies had certain limitations such as a small sample size and short follow-up time. In 2018, a phase 2 trial (RTOG 0618) reported SBRT (54 Gy in 3 fractions) achieved excellent efficacy and safety for operable stage I NSCLC (45). A recent trial (revised STARS) found that SABR was noninferior to surgery in treating operable stage I NSCLC, with 3-year OS and severe toxicity rates of 91% and 1%, respectively (46).

Some studies suggested that stereotactic body proton therapy (SBPT) had superior dosimetric features to photon based SBRT (47). In 2012, a retrospective study reported that SBPT is effective and well tolerated for inoperable stage I NSCLC (48). In 2018, a phase 2 randomized trial was conducted to compare SBPT and photon based SBRT for early-stage NSCLC. However, this trial closed early owing to poor accrual, largely because of insurance coverage and lack of volumetric imaging in the SBPT group (49). Some retrospective studies reported that SBPT (51—70 Gy in 10 fractions) for both central and peripheral early-stage NSCLC achieved excellent local control with minor toxicity (50, 51). A recently published retrospective study compared SBPT with SBRT for early-stage NSCLC. The results showed that patients who received either SBPT or SBRT achieved similar outcomes, although those who received SBPT had a higher risk of developing radiation pneumonitis (52).

SBRT has been a standard treatment for early-stage NSCLC in recent years. Several studies have validated the suitable dose fractions for tumors at different sites and demonstrated the noninferiority of SBRT to surgery for operable stage I NSCLC. The toxicity limited the dose, therefore also limited the local efficacy of SBRT. Induction systemic therapy might reduce tumor burden and extent, so as to reduce radiation toxicity. Distant recurrence after SBRT should be valued, concurrent or consolidated systemic therapy might reduce recurrent rate. Selected patients may benefit from SBPT rather than photon-based SBRT, but high-quality clinical evidence is lacking. Therefore, further clinical trials are needed to further established the optimal and individual treatment strategy for patients with early-stage NSCLC.

### Perioperative radiotherapy for NSCLC

Neoadjuvant radiotherapy and/or chemoradiotherapy may improve the prognosis of patients with stage III NSCLC. A phase 2 study conducted in 1995 was the first to assess the feasibility of preoperative CCRT, and reported a 3-year OS rate of 26% (53). However, in a randomized trial of patients with stage III NSCLC, preoperative chemoradiation in addition to chemotherapy did not improve OS (54). The treatment of patients with stage IIIA NSCLC with ipsilateral mediastinal nodal metastases (N2) is controversial, with a nonrandomized phase 3 trial demonstrating that CCRT with or without resection (preferably lobectomy) is a viable therapeutic option for patients with N2 nodal disease (54). Moreover, a phase 3 randomized trial demonstrated that radiotherapy did not add any benefit to induction chemotherapy followed by surgery for patients with N2 disease (55). Some studies neoadjuvant SBRT for early-stage NSCLC. In 2019, a phase 2 trial reported neoadjuvant SBRT yielded a pathological complete response rate as high as 60% (13).

Postoperative radiotherapy (PORT) can potentially reduce the rate of recurrence but at the expense of toxicity. Patients with incompletely resected NSCLC obviously benefited from PORT (56). A meta-analysis published in 1998 showed that PORT was detrimental to the outcome of patients with completely resected NSCLC (57). Whether patients with completely resected N2 disease benefit from PORT was controversial. A population-based cohort study from 2006 found that postoperative radiotherapy increased OS only in patients with N2 disease (58), which was confirmed by a retrospective analysis of a randomized trial (ANITA) (59). The data from a prospective nationwide oncology outcomes database suggested improved survival was associated with receipt of PORT for patients with completely resected N2 disease (60). However, a randomized phase 3 trial (PORT-C) reported that postoperative radiotherapy following complete resection and adjuvant chemotherapy did not improve disease-free survival (DFS) in patients with pIIIA-N2 NSCLC (61). A recently published retrospective analysis suggested that patients with radiation-resistance gene alterations may derive minimal benefit from PORT, whereas patients with a high tumor mutational burden and/or alterations in DNA damage response and repair genes may benefit from PORT (62). A propensity score matched analysis suggested patients with N2 squamous cell lung cancer benefited from PORT (63). Moreover, A machine learning-based model was developed to predict the prognosis of patients with N2 disease and suggested that patients with a high lymph node burden or lymph node ratio might benefit from PORT (64).

Perioperative adjuvant radiotherapy or chemoradiotherapy improves the prognosis of selected patients with NSCLC. The main controversy centers on the management of completely resected N2 NSCLC. Some subsets of patients are reported to benefit from PORT, but the clinical evidence is lacking. Clinical trials are needed to further identify the subset of patients that would derive the greatest clinical benefit from perioperative radiotherapy.

### Radiotherapy for NSCLC with driver gene alteration

Early studies suggested that the efficacy of radiotherapy was limited in patients with epidermal growth factor receptor (EGFR) or anaplastic lymphoma kinase (ALK) gene altered NSCLC (65). For these patients, TKIs were effective and safe, and the combination of radiotherapy and TKI seemed promising. However, some studies reported excessive toxicity of TKIs with concurrent chest or brain radiotherapy (66–68). Moreover, some of the other studies showed negative results of TKIs with concurrent or sequential radiotherapy (22, 25, 26). Clinical evidence for the combination of radiotherapy with TKIs is still lacking. A recent phase 3 randomized trial evaluated whole-brain radiotherapy with concurrent erlotinib in NSCLC with brain metastases (BM), however, erlotinib did not improve prognosis (69).

The combination of first- or second-generation TKIs and concurrent radiotherapy is not favored for patients with EGFR or ALK gene alteration. The optimal combination of radiotherapy and TKIs may be sequential. Currently, the efficacy of third-generation TKIs (eg. osimertinib and lorlatnib) has been proved. Radiotherapy plus third-generation TKIs may be beneficial, but high-quality clinical evidence is needed.

### Chemoradiotherapy for SCLC

Fewer studies have been conducted on radiotherapy for SCLC than for NSCLC. A meta-analysis published in 1992 concluded that radiotherapy improved OS in patients with limited stage (LS-)SCLC (70); and a randomized trial conducted in 1993 showed that CCRT was superior to sequential chemoradiotherapy for LS-SCLC (71). The optimal irradiation dose fraction was controversial. Hyperfractionated CCRT (radiation dose of 1.5 Gy twice a day) was shown to be superior to standard CCRT, and a shorter time between the first day of chemotherapy and last day of radiotherapy was associated with improved OS in L-SCLC (72). In 2017, the CONVERT phase 3 trial failed to prove the superiority of once-daily CCRT to twice-daily CCRT (73). However, a randomized phase 2 trial suggested moderately hypofractionated CCRT (60 Gy in 26 fractions) achieved longer PFS and similar toxicities compared with hyperfractionated CCRT (45 Gy in 30 fractions) (74). Moreover, another randomized phase 2 trial reported that twice-daily radiotherapy of 60 Gy in 40 fractions substantially improved survival compared to 45 Gy, without increased toxicity (75). The standard treatment for ES-SCLC used to be chemotherapy. In 2015, a phase 3 randomized trial demonstrated that the addition of thoracic radiotherapy improved the OS of patients with ES-SCLC who respond to chemotherapy (76).

Chemoradiotherapy is the standard treatment for LS-SCLC. However, the optimal dose fraction is still controversial. Thoracic radiotherapy may improve the prognosis of patients with ES-SCLC, but further studies is need to clarify the patient selection and treatment strategy. Moreover, clinical trials are ongoing to evaluate the combination of radiotherapy and ICIs for patients with SCLC (77, 78).

### Prophylactic cranial irradiation

BM is common and lethal in patients with SCLC. A randomized trial conducted in 1995 was the first to demonstrate that prophylactic cranial irradiation (PCI) for patients with LS-SCLC in complete remission (CR) decreased the risk of BM without a significant increase in complications (79). A meta-analysis published in 1999 concluded that PCI improved both OS and DFS in patients with L-SCLC in CR (80). The necessity of PCI for extensive (ES-)SCLC is controversial. A randomized trial from 2007 reported that PCI reduced the incidence of symptomatic BM and prolonged DFS and OS in ES-SCLC (81); however, another randomized phase 3 trial reported that PCI was not essential for patients with E-SCLC with response to initial chemotherapy and a confirmed absence of BM when patients receive periodic magnetic resonance imaging (MRI) examination during follow-up (82). PCI was previously considered unnecessary for patients with NSCLC based on a lack of OS benefit and treatment-associated memory decline (83). However, a recent randomized phase 2 trial (PRoT-BM) reported that PCI decreased the incidence of BM and prolonged PFS and OS in selected patients at high risk for developing BM (84). The major complication after PCI is cognitional functional disorder; a recent randomized phase 3 trial found that sparing the hippocampus during PCI preserved cognitive function while no differences were observed with respect to brain failure and OS compared to standard PCI (85).

PCI is still essential for LS-SCLC, but further studies are needed to identify the patients with NSCLC or ES-SCLC who would benefit from PCI. An optimized definition of target volume can improve patients’ quality of life.

### Radiotherapy plus ICIs

ICIs combined with radiotherapy has been one of the hottest areas in lung cancer research, despite the negative results reported by some studies (86, 87).

ICIs plus chemoradiotherapy further improved the prognosis of patients with locally advanced NSCLC. A randomized phase 3 trial (PACIFIC) showed that CCRT followed by durvalumab as consolidation therapy significantly prolonged progression-free survival (PFS) (10). The recently reported 5-year results further demonstrated the OS and PFS benefit with durvalumab after chemoradiotherapy (88). In addition, a recent real-world cohort study demonstrated the timing of durvalumab initiation up to 120 days after chemotherapy completion is not associated with the prognosis (89). However, another recent study suggested that the frequency of tumor-reactive CD8(+) T cells decreased after CCRT (90). Therefore, earlier administration of ICIs might further improve the efficacy compared with immunotherapy after CCRT (90). The efficacy of other ICIs, such as pembrolizumab and sugemalimab, combined with CCRT was also proved by clinical trials (KEYNOTE-799, GEMSTONE-301) (91, 92).

​There are fewer studies of radiotherapy plus ICIs for SCLC than for NSCLC. In 2020, a phase 1/2 trial suggested that pembrolizumab plus CCRT yielded favorable outcomes for patients with LS-SCLC (93). A phase 1 trial aimed to evaluate pembrolizumab plus radiotherapy after induction chemotherapy for ES-SCLC. However, this trial yielded no meaningful results due to heterogeneity in eligibility criteria (94). A recent randomized phase 2 trial (STIMULI) evaluated consolidation nivolumab and ipilimumab for patients with LS-SCLC after CCRT. However, this trial did not meet its primary endpoint of improving PFS with nivolumab-ipilimumab consolidation after CCRT in LS-SCLC (95).

SBRT may increase tumor antigen release, antigen presentation, and T-cell infiltration (96). A phase 2 trial (PEMBRO-RT) reported that additional SABR before pembrolizumab improved the efficacy for patients with metastatic NSCLC (96). Notably, patients without PD-L1 expression achieved a greater improvement in PFS and OS than others (96). A randomized phase 2 trial evaluating whether SBRT could enhance the effect of ICIs by increasing tumor response in nonirradiated metastatic NSCLC lesions showed that although there was a doubling of ORR, the results did not meet the study’s prespecified endpoint criteria for meaningful clinical benefit (96). In a randomized phase 2 trial, SBRT with neoadjuvant durvalumab resulted in a high pathologic response rate and was well-tolerated in early-stage NSCLC patients (97). The treatment for relapsed SCLC was challenging. A randomized phase 3 trial evaluated durvalumab and tremelimumab plus SBRT for relapsed SCLC, but the efficacy was disappointing (98). Multiple trials are ongoing to further evaluate SBRT plus ICIs for lung cancer (99–101).

​The synergistic effect of radiotherapy and ICIs has been demonstrated in both basic and clinical studies. The addition of ICIs to radiotherapy/chemoradiotherapy/SBRT has been shown to be effective in selected patients with NSCLC. However, evidence in favor of radiotherapy plus ICIs for SCLC is still lacking. Further clinical trials are ongoing to identify targeted patient populations and the most effective combination of treatments.

### Journals, countries, institutions, and authors

*Int. J. Radiat. Oncol. Biol. Phys.* was the most productive journal on lung cancer radiotherapy. *Int. J. Radiat. Oncol. Biol. Phys.* also had the highest local citation number, indicating that it is highly influential in this area. *J. Clin. Oncol.* was also both productive and influential, with 153 publications and an average citation per paper per year of 15.08. The paper numbers of *N. Engl. J. Med.* and *Lancet* were low, but these papers were very influential. Among the 28 journals with the top-papers, 20 were considered to be top-journals. The articles published in top-journals were likely to be impactful. Therefore, the analysis of top-journals could help researchers to identify the important recently published articles. In particular, most of the top-papers were published in comprehensive journals, which may be due to the high impact factors of these journals.

Corresponding authors from the USA and China mainland contributed nearly a half of the articles. However, articles by corresponding authors from the USA were much more influential than from China mainland. International collaboration was rare in Asian countries but common in American and European countries. Most authors of the top-papers were from developed countries/regions, and international collaboration of these articles was common. Currently, high-quality studies from developing countries/regions are still lacking. The most productive institution was the University of Texas MD Anderson Cancer Center. Although some universities in China mainland were productive, their TPRs were low. In contrast, although some other institutions did not publish many papers, they contributed to many top-papers (eg. Indiana University and National Yang Ming University). Komaki R was the most cited author in this area, and the most productive authors of the top-papers were Choy H, Senan S, and Nagata Y. As corresponding authors, Yu JM, Rades D, and Liao ZX were most productive, and Timmerman R, Antonia SJ, Bradley JD were most cited.

This study presents the most influential journals, countries, institutions, and authors on lung cancer radiotherapy and visualizes the collaboration networks. The results can help researchers select target journals for publication and find potential cooperative partners.

### Research trends and hotspots

This study quantitatively and comprehensively analyzes the research trends, status, and hotspots in lung cancer radiotherapy based on 9868 articles published between 2000 and 2022. Other major review methods, such as systematic literature review and meta-analysis, are unapplicable for this purpose (8).

This study analyzed and visualized the research trends on lung cancer radiotherapy. Chemoradiotherapy was always the hottest research area, and the number of papers on metastatic lung cancer or PET/CT gradually increased. The number of publications on preoperative radiotherapy, postoperative radiotherapy, and PCI varied little from year to year. SBRT for lung cancer has been a research hotspot since 2006, and radiotherapy plus immunotherapy has been highly focused since 2019.

The current status on lung cancer radiotherapy is: 1) chemoradiotherapy is the standard treatment for advanced lung cancer; 2) neoadjuvant/adjuvant radiotherapy prolongs the OS in selected patients; 3) SBRT is not inferior to surgery for early-stage NSCLC; 4) PCI is necessary in patients with LS-SCLC; 5) selected patients with metastatic lung cancer benefit from radiotherapy; and 6) CCRT plus anti-PD-1 or anti-PD-L1 antibodies can further improve the prognosis of patients with advanced lung cancer.

The current research hotspots include: 1) the optimal dose fraction of CCRT for SCLC; 2) the necessity of neoadjuvant/adjuvant radiotherapy for patients with N2 disease; 3) SBRT for early-stage NSCLC; 4) management of SCLC brain metastases based on MRI; 5) radiotherapy for lung cancer oligometastasis; and 6) neoadjuvant radiotherapy plus ICIs. The authors suggest that important future research directions include: 1) SBRT plus ICIs for lung cancer oligometastasis; 2) radiotherapy plus ICIs for SCLC; 3) individualized treatment for special patients; 4) radiotherapy plus novel ICIs; 5) the mechanisms of radiation resistance; and 6) radiomics based on CT, MRI, and PET.

### Limitations

The bibliometric analysis described herein has certain limitations. 1) This study aims to present the landscape of radiotherapy for lung cancer, and only includes relevant articles published between 2000 and 2022. Thus, earlier articles are excluded. 2) Due to the large number of articles, it is impossible to read every article and respectively analyze the subareas. In order to better present the research trends and status of the subareas, the authors discuss the developments and recent advances of subareas. 3) This study focuses on clinical studies and the search strategy may omit the important basic research studies, and the authors do not discuss radiobiology as well as radiophysics. 4) Finally, the authors conduct the literature retrieval only based on the Web of Science (Science Citation Indexing Expanded database), and articles not included in this database are omitted. This may lead to selection bias and analytical errors.

## Conclusion

To our knowledge, this study is the first comprehensive and quantitative bibliometric analysis of lung cancer radiotherapy. This study demonstrates the research trends and hotspots based on an analysis of 9868 articles and 100 top-papers. Moreover, the results can help researchers in selecting target journals for publication and in findings potential collaborators. The authors suggest that important research directions include: 1) SBRT plus ICIs for lung cancer oligometastasis; 2) radiotherapy plus ICIs for SCLC; 3) individualized treatment for special patients; 4) radiotherapy plus novel ICIs; 5) the mechanisms of radiation resistance; and 6) radiomics based on CT, MRI, and PET. This study can help researchers gain a comprehensive picture of the research landscape, historical development, and recent hotspots in lung cancer radiotherapy and can provide inspiration for future research.

## Data availability statement

The raw data supporting the conclusions of this article will be made available by the authors, without undue reservation.

## Author contributions

XZ and YHL contributed to the study's conception. YHL analyzed the data. YHL, SJ, YRL, HY, and LY contributed to the literature review. YHL wrote the manuscript. All authors contributed to the article and approved the submitted version.

## Conflict of interest

The authors declare that the research was conducted in the absence of any commercial or financial relationships that could be construed as a potential conflict of interest.

## Publisher’s note

All claims expressed in this article are solely those of the authors and do not necessarily represent those of their affiliated organizations, or those of the publisher, the editors and the reviewers. Any product that may be evaluated in this article, or claim that may be made by its manufacturer, is not guaranteed or endorsed by the publisher.

## Supplementary material

Supplementary Material S1 Detailed search strategy for the papers on lung cancer radiotherapy.

Supplementary Table S1 The 100 top-papers on lung cancer radiotherapy.

Supplementary Table S2 The journals with top-papers on lung cancer radiotherapy.

Supplementary Table S3 The papers on lung cancer radiotherapy published in major journals since 2020.

Supplementary Table S4 The corresponding authors with at least two top-papers in lung cancer radiotherapy from 2000 to 2022.

Supplementary Figure 1 Historical direct citation network among the publications.

Supplementary Figure 2 Top 50 references with the strongest citation bursts on lung cancer radiotherapy.

Supplementary Figure 3 Citation relationship network of the publications with the strongest link strength.

Supplementary Figure 4 The trends of the keywords rank of lung cancer radiotherapy.

Supplementary Figure 5 Top 50 keywords with the strongest citation bursts on lung cancer radiotherapy.

## References

[1] Sung H Ferlay J Siegel RL Laversanne M Soerjomataram I Jemal A. Global Cancer Statistics 2020: GLOBOCAN Estimates of Incidence and Mortality Worldwide for 36 Cancers in 185 Countries. CA Cancer J Clin (2021) 71(3):209–49. doi: 10.3322/caac.21660 33538338

[2] Duma N Santana-Davila R Molina JR. Non-small cell lung cancer: Epidemiology, screening, diagnosis, and treatment. Mayo Clin Proc (2019) 94:1623–40. doi: 10.1016/j.mayocp.2019.01.013 31378236

[3] Cox JD Azarnia N Byhardt RW Shin KH Emami B Pajak TF. A randomized phase I/II trial of hyperfractionated radiation therapy with total doses of 60.0 gy to 79.2 gy: Possible survival benefit with greater than or equal to 69.6 gy in favorable patients with radiation therapy oncology group stage III non-small-cell lung carcinoma: Report of radiation therapy oncology group 83-11. J Clin Oncol (1990) 8:1543–55. doi: 10.1200/JCO.1990.8.9.1543 2167952

[4] Le Chevalier T Arriagada R Quoix E Ruffie P Martin M Tarayre M. Radiotherapy alone versus combined chemotherapy and radiotherapy in nonresectable non-small-cell lung cancer: First analysis of a randomized trial in 353 patients. J Natl Cancer Inst (1991) 83:417–23. doi: 10.1093/jnci/83.6.417 1847977

[5] Nagata Y Takayama K Matsuo Y Norihisa Y Mizowaki T Sakamoto T. Clinical outcomes of a phase I/II study of 48 gy of stereotactic body radiotherapy in 4 fractions for primary lung cancer using a stereotactic body frame. Int J Radiat Oncol Biol Phys (2005) 63:1427–31. doi: 10.1016/j.ijrobp.2005.05.034 16169670

[6] Liu Y Xu Y Cheng X Lin Y Jiang S Yu H. Research trends and most influential clinical studies on anti-PD1/PDL1 immunotherapy for cancers: A bibliometric analysis. Front Immunol (2022) 13:862084. doi: 10.3389/fimmu.2022.862084 35493449 PMC9044908

[7] Liu Y Li J Cheng X Zhang X. Bibliometric analysis of the top-cited publications and research trends for stereotactic body radiotherapy. Front Oncol (2021) 11:795568. doi: 10.3389/fonc.2021.795568 34926312 PMC8677697

[8] Donthu N Kumar S Mukherjee D Pandey N Lim WM. How to conduct a bibliometric analysis: An overview and guidelines. J Of Business Res (2021) 133:285–96. doi: 10.1016/j.jbusres.2021.04.070

[9] Yeung AWK. Comparison between scopus, web of science, PubMed and publishers for mislabelled review papers. Curr Sci India. (2019) 116:1909–14. doi: 10.1186/s13104-016-2026-2

[10] Antonia SJ Villegas A Daniel D Vicente D Murakami S Hui R. Durvalumab after chemoradiotherapy in stage III non-Small-Cell lung cancer. N Engl J Med (2017) 377:1919–29. doi: 10.1056/NEJMoa1709937 28885881

[11] Timmerman R Paulus R Galvin J Michalski J Straube W Bradley J. Stereotactic body radiation therapy for inoperable early stage lung cancer. JAMA. (2010) 303:1070–6. doi: 10.1001/jama.2010.261 PMC2907644 20233825

[12] Paz-Ares L Dvorkin M Chen Y Reinmuth N Hotta K Trukhin D. Durvalumab plus platinum-etoposide versus platinum-etoposide in first-line treatment of extensive-stage small-cell lung cancer (CASPIAN): A randomised, controlled, open-label, phase 3 trial. Lancet. (2019) 394:1929–39. doi: 10.1016/S0140-6736(19)32222-6 31590988

[13] Palma DA Nguyen TK Louie AV Malthaner R Fortin D Rodrigues GB. Measuring the integration of stereotactic ablative radiotherapy plus surgery for early-stage non-small cell lung cancer: A phase 2 clinical trial. JAMA Oncol (2019) 5:681–8. doi: 10.1001/jamaoncol.2018.6993 PMC6512269 30789648

[14] Verellen D Linthout N van den Berge D Bel A Storme G. Initial experience with intensity-modulated conformal radiation therapy for treatment of the head and neck region. Int J Radiat Oncol Biol Phys (1997) 39:99–114. doi: 10.1016/s0360-3016(97)00304-0 9300745

[15] van Herk M Bruce A Kroes AP Shouman T Touw A Lebesque JV. Quantification of organ motion during conformal radiotherapy of the prostate by three dimensional image registration. Int J Radiat Oncol Biol Phys (1995) 33:1311–20. doi: 10.1016/0360-3016(95)00116-6 7493856

[16] Dillman RO Seagren SL Propert KJ Guerra J Eaton WL Perry MC. A randomized trial of induction chemotherapy plus high-dose radiation versus radiation alone in stage III non-small-cell lung cancer. N Engl J Med (1990) 323:940–5. doi: 10.1056/NEJM199010043231403 2169587

[17] Schaake-Koning C van den Bogaert W Dalesio O Festen J Hoogenhout J van Houtte P. Effects of concomitant cisplatin and radiotherapy on inoperable non-small-cell lung cancer. N Engl J Med (1992) 326:524–30. doi: 10.1056/NEJM199202203260805 1310160

[18] Sause WT Scott C Taylor S Johnson D Livingston R Komaki R. Radiation therapy oncology group (RTOG) 88-08 and Eastern cooperative oncology group (ECOG) 4588: Preliminary results of a phase III trial in regionally advanced, unresectable non-small-cell lung cancer. J Natl Cancer Inst (1995) 87:198–205. doi: 10.1093/jnci/87.3.198 7707407

[19] Furuse K Fukuoka M Kawahara M Nishikawa H Takada Y Kudoh S. Phase III study of concurrent versus sequential thoracic radiotherapy in combination with mitomycin, vindesine, and cisplatin in unresectable stage III non-small-cell lung cancer. J Clin Oncol (1999) 17:2692–9. doi: 10.1200/JCO.1999.17.9.2692 10561343

[20] Zatloukal P Petruzelka L Zemanova M Havel L Janku F Judas L. Concurrent versus sequential chemoradiotherapy with cisplatin and vinorelbine in locally advanced non-small cell lung cancer: A randomized study. Lung Cancer. (2004) 46:87–98. doi: 10.1016/j.lungcan.2004.03.004 15364136

[21] Fournel P Robinet G Thomas P Souquet PJ Léna H Vergnenégre A. Randomized phase III trial of sequential chemoradiotherapy compared with concurrent chemoradiotherapy in locally advanced non-small-cell lung cancer: Groupe Lyon-Saint-Etienne d'Oncologie thoracique-groupe français de pneumo-cancérologie NPC 95-01 study. J Clin Oncol (2005) 23:5910–7. doi: 10.1200/JCO.2005.03.070 16087956

[22] Kelly K Chansky K Gaspar LE Albain KS Jett J Ung YC. Phase III trial of maintenance gefitinib or placebo after concurrent chemoradiotherapy and docetaxel consolidation in inoperable stage III non-small-cell lung cancer: SWOG S0023. J Clin Oncol (2008) 26:2450–6. doi: 10.1200/JCO.2007.14.4824 18378568

[23] Vokes EE Herndon JE 2nd Kelley MJ Cicchetti MG Ramnath N Neill H. Induction chemotherapy followed by chemoradiotherapy compared with chemoradiotherapy alone for regionally advanced unresectable stage III non-small-cell lung cancer: Cancer and leukemia group b. J Clin Oncol (2007) 25:1698–704. doi: 10.1200/JCO.2006.07.3569 17404369

[24] Hanna N Neubauer M Yiannoutsos C McGarry R Arseneau J Ansari R. Phase III study of cisplatin, etoposide, and concurrent chest radiation with or without consolidation docetaxel in patients with inoperable stage III non-small-cell lung cancer: the Hoosier oncology group and U.S. oncology. J Clin Oncol (2008) 26:5755–60. doi: 10.1200/JCO.2008.17.7840 19001323

[25] Bradley JD Paulus R Komaki R Masters G Blumenschein G Schild S. Standard-dose versus high-dose conformal radiotherapy with concurrent and consolidation carboplatin plus paclitaxel with or without cetuximab for patients with stage IIIA or IIIB non-small-cell lung cancer (RTOG 0617): A randomised, two-by-two factorial phase 3 study. Lancet Oncol (2015) 16:187–99. doi: 10.1016/S1470-2045(14)71207-0 PMC4419359 25601342

[26] Bradley JD Hu C Komaki RR Masters GA Blumenschein GR Schild SE. Long-term results of NRG oncology RTOG 0617: Standard- versus high-dose chemoradiotherapy with or without cetuximab for unresectable stage III non-Small-Cell lung cancer. J Clin Oncol (2020) 38:706–14. doi: 10.1200/JCO.19.01162 PMC7048161 31841363

[27] Skinner H Hu C Tsakiridis T Santana-Davila R Lu B Erasmus JJ. Addition of metformin to concurrent chemoradiation in patients with locally advanced non-small cell lung cancer: The NRG-LU001 phase 2 randomized clinical trial. JAMA Oncol (2021) 7:1324–32. doi: 10.1001/jamaoncol.2021.2318 PMC8323052 34323922

[28] Shioyama Y Tokuuye K Okumura T Kagei K Sugahara S Ohara K. Clinical evaluation of proton radiotherapy for non-small-cell lung cancer. Int J Radiat Oncol Biol Phys (2003) 56:7–13. doi: 10.1016/s0360-3016(02)04416-4 12694818

[29] Sejpal S Komaki R Tsao A Chang JY Liao Z Wei X. Early findings on toxicity of proton beam therapy with concurrent chemotherapy for nonsmall cell lung cancer. Cancer. (2011) 117:3004–13. doi: 10.1002/cncr.25848 21264827

[30] Chang JY Komaki R Lu C Wen HY Allen PK Tsao A. Phase 2 study of high-dose proton therapy with concurrent chemotherapy for unresectable stage III nonsmall cell lung cancer. Cancer. (2011) 117:4707–13. doi: 10.1002/cncr.26080 PMC3174272 21437893

[31] Higgins KA O'Connell K Liu Y Gillespie TW McDonald MW Pillai RN. National cancer database analysis of proton versus photon radiation therapy in non-small cell lung cancer. Int J Radiat Oncol Biol Phys (2017) 97:128–37. doi: 10.1016/j.ijrobp.2016.10.001 27979443

[32] Liao Z Lee JJ Komaki R Gomez DR O'Reilly MS Fossella FV. Bayesian Adaptive randomization trial of passive scattering proton therapy and intensity-modulated photon radiotherapy for locally advanced non-Small-Cell lung cancer. J Clin Oncol (2018) 36:1813–22. doi: 10.1200/JCO.2017.74.0720 PMC6008104 29293386

[33] Hoppe BS Nichols RC Flampouri S Pankuch M Morris CG Pham DC. Chemoradiation with hypofractionated proton therapy in stage II-III non-small cell lung cancer: A proton collaborative group phase 2 trial. Int J Radiat Oncol Biol Phys (2022) 113:732–41. doi: 10.1016/j.ijrobp.2022.03.005 35306151

[34] Contreras J Srivastava A Samson P DeWees T Govindan R Baggstrom MQ. Phase I study of accelerated hypofractionated proton therapy and chemotherapy for locally advanced non-small cell lung cancer. Int J Radiat Oncol Biol Phys (2022) 113:742–8. doi: 10.1016/j.ijrobp.2022.01.012 35074432

[35] Ball D Mai GT Vinod S Babington S Ruben J Kron T. Stereotactic ablative radiotherapy versus standard radiotherapy in stage 1 non-small-cell lung cancer (TROG 09.02 CHISEL): A phase 3, open-label, randomised controlled trial. Lancet Oncol (2019) 20:494–503. doi: 10.1016/S1470-2045(18)30896-9 30770291

[36] Senthi S Lagerwaard FJ Haasbeek CJ Slotman BJ Senan S. Patterns of disease recurrence after stereotactic ablative radiotherapy for early stage non-small-cell lung cancer: A retrospective analysis. Lancet Oncol (2012) 13:802–9. doi: 10.1016/S1470-2045(12)70242-5 22727222

[37] Timmerman R McGarry R Yiannoutsos C Papiez L Tudor K DeLuca J. Excessive toxicity when treating central tumors in a phase II study of stereotactic body radiation therapy for medically inoperable early-stage lung cancer. J Clin Oncol (2006) 24:4833–9. doi: 10.1200/JCO.2006.07.5937 17050868

[38] Haasbeek CJ Lagerwaard FJ Slotman BJ Senan S. Outcomes of stereotactic ablative radiotherapy for centrally located early-stage lung cancer. J Thorac Oncol (2011) 6:2036–43. doi: 10.1097/JTO.0b013e31822e71d8 21892102

[39] Chang JY Li QQ Xu QY Allen PK Rebueno N Gomez DR. Stereotactic ablative radiation therapy for centrally located early stage or isolated parenchymal recurrences of non-small cell lung cancer: How to fly in a "no fly zone". Int J Radiat Oncol Biol Phys (2014) 88:1120–8. doi: 10.1016/j.ijrobp.2014.01.022 24661665

[40] Chang JY Balter PA Dong L Yang Q Liao Z Jeter M. Stereotactic body radiation therapy in centrally and superiorly located stage I or isolated recurrent non-small-cell lung cancer. Int J Radiat Oncol Biol Phys (2008) 72:967–71. doi: 10.1016/j.ijrobp.2008.08.001 PMC5113613 18954709

[41] Videtic GM Paulus R Singh AK Chang JY Parker W Olivier KR. Long-term follow-up on NRG oncology RTOG 0915 (NCCTG N0927): A randomized phase 2 study comparing 2 stereotactic body radiation therapy schedules for medically inoperable patients with stage I peripheral non-small cell lung cancer. Int J Radiat Oncol Biol Phys (2019) 103:1077–84. doi: 10.1016/j.ijrobp.2018.11.051 PMC6454873 30513377

[42] Verstegen NE Oosterhuis JW Palma DA Rodrigues G Lagerwaard FJ van der Elst A. Stage I-II non-small-cell lung cancer treated using either stereotactic ablative radiotherapy (SABR) or lobectomy by video-assisted thoracoscopic surgery (VATS): outcomes of a propensity score-matched analysis. Ann Oncol (2013) 24:1543–8. doi: 10.1093/annonc/mdt026 23425947

[43] Crabtree TD Denlinger CE Meyers BF El Naqa I Zoole J Krupnick AS. Stereotactic body radiation therapy versus surgical resection for stage I non-small cell lung cancer. J Thorac Cardiovasc Surg (2010) 140:377–86. doi: 10.1016/j.jtcvs.2009.12.054 20400121

[44] Chang JY Senan S Paul MA Mehran RJ Louie AV Balter P. Stereotactic ablative radiotherapy versus lobectomy for operable stage I non-small-cell lung cancer: A pooled analysis of two randomised trials. Lancet Oncol (2015) 16:630–7. doi: 10.1016/S1470-2045(15)70168-3 PMC4489408 25981812

[45] Timmerman RD Paulus R Pass HI Gore EM Edelman MJ Galvin J. Stereotactic body radiation therapy for operable early-stage lung cancer: Findings from the NRG oncology RTOG 0618 trial. JAMA Oncol (2018) 4:1263–6. doi: 10.1001/jamaoncol.2018.1251 PMC6117102 29852037

[46] Chang JY Mehran RJ Feng L Verma V Liao Z Welsh JW. Stereotactic ablative radiotherapy for operable stage I non-small-cell lung cancer (revised STARS): Long-term results of a single-arm, prospective trial with prespecified comparison to surgery. Lancet Oncol (2021) 22:1448–57. doi: 10.1016/S1470-2045(21)00401-0 PMC8521627 34529930

[47] Hoppe BS Huh S Flampouri S Nichols RC Oliver KR Morris CG. Double-scattered proton-based stereotactic body radiotherapy for stage I lung cancer: A dosimetric comparison with photon-based stereotactic body radiotherapy. Radiother Oncol (2010) 97:425–30. doi: 10.1016/j.radonc.2010.09.006 20934768

[48] Westover KD Seco J Adams JA Lanuti M Choi NC Engelsman M. Proton SBRT for medically inoperable stage I NSCLC. J Thorac Oncol (2012) 7:1021–5. doi: 10.1097/JTO.0b013e31824de0bf PMC3354010 22551902

[49] Nantavithya C Gomez DR Wei X Komaki R Liao Z Lin SH. Phase 2 study of stereotactic body radiation therapy and stereotactic body proton therapy for high-risk, medically inoperable, early-stage non-small cell lung cancer. Int J Radiat Oncol Biol Phys (2018) 101:558–63. doi: 10.1016/j.ijrobp.2018.02.022 29680255

[50] Kharod SM Nichols RC Henderson RH Morris CG Pham DC Seeram VK. Image-guided hypofractionated double-scattering proton therapy in the management of centrally-located early-stage non-small cell lung cancer. Acta Oncol (2020) 59:1164–70. doi: 10.1080/0284186X.2020.1759821 32394776

[51] Bush DA Cheek G Zaheer S Wallen J Mirshahidi H Katerelos A. High-dose hypofractionated proton beam radiation therapy is safe and effective for central and peripheral early-stage non-small cell lung cancer: results of a 12-year experience at Loma Linda University Medical Center. Int J Radiat Oncol Biol Phys (2013) 86:964–8. doi: 10.1016/j.ijrobp.2013.05.002 23845845

[52] Bae BK Yang K Noh JM Pyo H Ahn YC. Clinical outcomes following proton and photon stereotactic body radiation therapy for early-stage lung cancer. Cancers (Basel). (2022) 14. doi: 10.3390/cancers14174152 PMC9454659 36077688

[53] Albain KS Rusch VW Crowley JJ Rice TW Turrisi AT 3rd Weick JK. Concurrent cisplatin/etoposide plus chest radiotherapy followed by surgery for stages IIIA (N2) and IIIB non-small-cell lung cancer: Mature results of southwest oncology group phase II study 8805. J Clin Oncol (1995) 13:1880–92. doi: 10.1200/JCO.1995.13.8.1880 7636530

[54] Thomas M Rübe C Hoffknecht P Macha HN Freitag L Linder A. Effect of preoperative chemoradiation in addition to preoperative chemotherapy: A randomised trial in stage III non-small-cell lung cancer. Lancet Oncol (2008) 9:636–48. doi: 10.1016/S1470-2045(08)70156-6 18583190

[55] Pless M Stupp R Ris HB Stahel RA Weder W Thierstein S. Induction chemoradiation in stage IIIA/N2 non-small-cell lung cancer: A phase 3 randomised trial. Lancet. (2015) 386:1049–56. doi: 10.1016/S0140-6736(15)60294-X 26275735

[56] Wang EH Corso CD Rutter CE Park HS Chen AB Kim AW. Postoperative radiation therapy is associated with improved overall survival in incompletely resected stage II and III non-Small-Cell lung cancer. J Clin Oncol (2015) 33:2727–34. doi: 10.1200/JCO.2015.61.1517 26101240

[57] PORT Meta-analysis Trialists Group. Postoperative radiotherapy in non-small-cell lung cancer: Systematic review and meta-analysis of individual patient data from nine randomised controlled trials. PORT meta-analysis trialists group. Lancet (1998) 352:257–63. doi: 10.1016/S0140-6736(98)06341-7 9690404

[58] Lally BE Zelterman D Colasanto JM Haffty BG Detterbeck FC Wilson LD. Postoperative radiotherapy for stage II or III non-small-cell lung cancer using the surveillance, epidemiology, and end results database. J Clin Oncol (2006) 24:2998–3006. doi: 10.1200/JCO.2005.04.6110 16769986

[59] Douillard JY Rosell R De Lena M Riggi M Hurteloup P Mahe MA. Impact of postoperative radiation therapy on survival in patients with complete resection and stage I, II, or IIIA non-small-cell lung cancer treated with adjuvant chemotherapy: The adjuvant navelbine international trialist association (ANITA) randomized trial. Int J Radiat Oncol Biol Phys (2008) 72:695–701. doi: 10.1016/j.ijrobp.2008.01.044 18439766

[60] Herskovic A Mauer E Christos P Nagar H. Role of postoperative radiotherapy in pathologic stage IIIA (N2) non-small cell lung cancer in a prospective nationwide oncology outcomes database. J Thorac Oncol (2017) 12:302–13. doi: 10.1016/j.jtho.2016.09.135 27746190

[61] Hui Z Men Y Hu C Kang J Sun X Bi N. Effect of postoperative radiotherapy for patients with pIIIA-N2 non-small cell lung cancer after complete resection and adjuvant chemotherapy: The phase 3 PORT-c randomized clinical trial. JAMA Oncol (2021) 7:1178–85. doi: 10.1001/jamaoncol.2021.1910 PMC8227450 34165501

[62] Shaverdian N Shepherd AF Li X Offin M Lengel HB Gelblum DY. Effects of tumor mutational burden and gene alterations associated with radiation response on outcomes of postoperative radiation therapy in non-small cell lung cancer. Int J Radiat Oncol Biol Phys (2022) 113:335–44. doi: 10.1016/j.ijrobp.2022.02.014 PMC9976944 35157996

[63] Wang Y Cao Y Wu M Lu Y He B Zhou L. Effect of preoperative radiotherapy on overall survival in N2 non-small-cell lung cancer: A propensity score-matched analysis of surveillance, epidemiology, and end results database. Interact Cardiovasc Thorac Surg (2022) 35. doi: 10.1093/icvts/ivab321 PMC9252120 35639970

[64] Zarinshenas R Ladbury C McGee H Raz D Erhunmwunsee L Pathak R. Machine learning to refine prognostic and predictive nodal burden thresholds for post-operative radiotherapy in completely resected stage III-N2 non-small cell lung cancer. Radiother Oncol (2022) 173:10–8. doi: 10.1016/j.radonc.2022.05.019 35618098

[65] Hayashi H Okamoto I Kimura H Sakai K Nishimura Y Nishio K. Clinical outcomes of thoracic radiotherapy for locally advanced NSCLC with EGFR mutations or EML4-ALK rearrangement. Anticancer Res (2012) 32:4533–7. 23060582

[66] Okamoto I Takahashi T Okamoto H Nakagawa K Watanabe K Nakamatsu K. Single-agent gefitinib with concurrent radiotherapy for locally advanced non-small cell lung cancer harboring mutations of the epidermal growth factor receptor. Lung Cancer (2011) 72:199–204. doi: 10.1016/j.lungcan.2010.08.016 20828860

[67] Kroeze SG Fritz C Hoyer M Lo SS Ricardi U Sahgal A. Toxicity of concurrent stereotactic radiotherapy and targeted therapy or immunotherapy: A systematic review. Cancer Treat Rev (2017) 53:25–37. doi: 10.1016/j.ctrv.2016.11.013 28056412

[68] Jiang T Min W Li Y Yue Z Wu C Zhou C. Radiotherapy plus EGFR TKIs in non-small cell lung cancer patients with brain metastases: An update meta-analysis. Cancer Med (2016) 5:1055–65. doi: 10.1002/cam4.673 PMC4924363 26990668

[69] Yang Z Zhang Y Li R Yisikandaer A Ren B Sun J. Whole-brain radiotherapy with and without concurrent erlotinib in NSCLC with brain metastases: A multicenter, open-label, randomized, controlled phase III trial. Neuro Oncol (2021) 23:967–78. doi: 10.1093/neuonc/noaa281 PMC8168818 33331923

[70] Pignon JP Arriagada R Ihde DC Johnson DH Perry MC Souhami RL. A meta-analysis of thoracic radiotherapy for small-cell lung cancer. N Engl J Med (1992) 327:1618–24. doi: 10.1056/NEJM199212033272302 1331787

[71] Murray N Coy P Pater JL Hodson I Arnold A Zee BC. Importance of timing for thoracic irradiation in the combined modality treatment of limited-stage small-cell lung cancer. Natl Cancer Institute Canada Clin Trials Group J Clin Oncol (1993) 11:336–44. doi: 10.1200/JCO.1993.11.2.336 8381164

[72] Turrisi AT 3rd Kim K Blum R Sause WT Livingston RB Komaki R. Twice-daily compared with once-daily thoracic radiotherapy in limited small-cell lung cancer treated concurrently with cisplatin and etoposide. N Engl J Med (1999) 340:265–71. doi: 10.1056/NEJM199901283400403 9920950

[73] Faivre-Finn C Snee M Ashcroft L Appel W Barlesi F Bhatnagar A. Concurrent once-daily versus twice-daily chemoradiotherapy in patients with limited-stage small-cell lung cancer (CONVERT): An open-label, phase 3, randomised, superiority trial. Lancet Oncol (2017) 18:1116–25. doi: 10.1016/S1470-2045(17)30318-2 PMC5555437 28642008

[74] Qiu B Li Q Liu J Huang Y Pang Q Zhu Z. Moderately hypofractionated once-daily compared with twice-daily thoracic radiation therapy concurrently with etoposide and cisplatin in limited-stage small cell lung cancer: A multicenter, phase II, randomized trial. Int J Radiat Oncol Biol Phys (2021) 111:424–35. doi: 10.1016/j.ijrobp.2021.05.003 33992717

[75] Grønberg BH Killingberg KT Fløtten ØVerifytat Brustugun OT Hornslien K Madebo T. High-dose versus standard-dose twice-daily thoracic radiotherapy for patients with limited stage small-cell lung cancer: An open-label, randomised, phase 2 trial. Lancet Oncol (2021) 22:321–31. doi: 10.1016/S1470-2045(20)30742-7 33662285

[76] Slotman BJ van Tinteren H Praag JO Knegjens JL El Sharouni SY Hatton M. Use of thoracic radiotherapy for extensive stage small-cell lung cancer: A phase 3 randomised controlled trial. Lancet. (2015) 385:36–42. doi: 10.1016/S0140-6736(14)61085-0 25230595

[77] Bozorgmehr F Christopoulos P Chung I Cvetkovic J Feißt M Krisam J. Protocol of the TREASURE study: Thoracic RadiothErapy with atezolizumab in small cell lUng canceR extensive disease - a randomized, open-label, multicenter phase II trial. BMC Cancer (2022) 22:1011. doi: 10.1186/s12885-022-10074-9 36153496 PMC9509547

[78] Senan S Okamoto I Lee GW Chen Y Niho S Mak G. Design and rationale for a phase III, randomized, placebo-controlled trial of durvalumab with or without tremelimumab after concurrent chemoradiotherapy for patients with limited-stage small-cell lung cancer: The ADRIATIC study. Clin Lung Cancer (2020) 21:e84–84e88. doi: 10.1016/j.cllc.2019.12.006 31948903

[79] Arriagada R Le Chevalier T Borie F Rivière A Chomy P Monnet I. Prophylactic cranial irradiation for patients with small-cell lung cancer in complete remission. J Natl Cancer Inst (1995) 87:183–90. doi: 10.1093/jnci/87.3.183 7707405

[80] Aupérin A Arriagada R Pignon JP Le Péchoux C Gregor A Stephens RJ. Prophylactic cranial irradiation for patients with small-cell lung cancer in complete remission. prophylactic cranial irradiation overview collaborative group. N Engl J Med (1999) 341:476–84. doi: 10.1056/NEJM199908123410703 10441603

[81] Slotman B Faivre-Finn C Kramer G Rankin E Snee M Hatton M. Prophylactic cranial irradiation in extensive small-cell lung cancer. N Engl J Med (2007) 357:664–72. doi: 10.1056/NEJMoa071780 17699816

[82] Takahashi T Yamanaka T Seto T Harada H Nokihara H Saka H. Prophylactic cranial irradiation versus observation in patients with extensive-disease small-cell lung cancer: A multicentre, randomised, open-label, phase 3 trial. Lancet Oncol (2017) 18:663–71. doi: 10.1016/S1470-2045(17)30230-9 28343976

[83] Sun A Bae K Gore EM Movsas B Wong SJ Meyers CA. Phase III trial of prophylactic cranial irradiation compared with observation in patients with locally advanced non-small-cell lung cancer: Neurocognitive and quality-of-life analysis. J Clin Oncol (2011) 29:279–86. doi: 10.1200/JCO.2010.29.6053 PMC3056463 21135267

[84] Arrieta O Maldonado F Turcott JG Zatarain-Barrón ZL Barrón F Blake-Cerda M. Prophylactic cranial irradiation reduces brain metastases and improves overall survival in high-risk metastatic non-small cell lung cancer patients: A randomized phase 2 study (PRoT-BM trial). Int J Radiat Oncol Biol Phys (2021) 110:1442–50. doi: 10.1016/j.ijrobp.2021.02.044 33640422

[85] Rodríguez de Dios N Couñago F Murcia-Mejía M Rico-Oses M Calvo-Crespo P Samper P. Randomized phase III trial of prophylactic cranial irradiation with or without hippocampal avoidance for small-cell lung cancer (PREMER): A GICOR-GOECP-SEOR study. J Clin Oncol (2021) 39:3118–27. doi: 10.1200/JCO.21.00639 34379442

[86] Formenti SC Rudqvist NP Golden E Cooper B Wennerberg E Lhuillier C. Radiotherapy induces responses of lung cancer to CTLA-4 blockade. Nat Med (2018) 24:1845–51. doi: 10.1038/s41591-018-0232-2 PMC6286242 30397353

[87] Butts C Socinski MA Mitchell PL Thatcher N Havel L Krzakowski M. Tecemotide (L-BLP25) versus placebo after chemoradiotherapy for stage III non-small-cell lung cancer (START): A randomised, double-blind, phase 3 trial. Lancet Oncol (2014) 15:59–68. doi: 10.1016/S1470-2045(13)70510-2 24331154

[88] Spigel DR Faivre-Finn C Gray JE Vicente D Planchard D Paz-Ares L. Five-year survival outcomes from the PACIFIC trial: Durvalumab after chemoradiotherapy in stage III non-Small-Cell lung cancer. J Clin Oncol (2022) 40:1301–11. doi: 10.1200/JCO.21.01308 PMC9015199 35108059

[89] Bryant AK Sankar K Strohbehn GW Zhao L Elliott D Daniel V. Timing of adjuvant durvalumab initiation is not associated with outcomes in stage III non-small cell lung cancer. Int J Radiat Oncol Biol Phys (2022) 113:60–5. doi: 10.1016/j.ijrobp.2021.12.176 PMC9018488 35115216

[90] Kim KH Pyo H Lee H Oh D Noh JM Ahn YC. Dynamics of circulating immune cells during chemoradiotherapy in patients with non-small cell lung cancer support earlier administration of anti-PD-1/PD-L1 therapy. Int J Radiat Oncol Biol Phys (2022) 113:415–25. doi: 10.1016/j.ijrobp.2022.02.003 35150786

[91] Jabbour SK Lee KH Frost N Breder V Kowalski DM Pollock T. Pembrolizumab plus concurrent chemoradiation therapy in patients with unresectable, locally advanced, stage III non-small cell lung cancer: The phase 2 KEYNOTE-799 nonrandomized trial. JAMA Oncol (2021) 7:1–9. doi: 10.1001/jamaoncol.2021.2301 PMC8446818 34086039

[92] Zhou Q Chen M Jiang O Pan Y Hu D Lin Q. Sugemalimab versus placebo after concurrent or sequential chemoradiotherapy in patients with locally advanced, unresectable, stage III non-small-cell lung cancer in China (GEMSTONE-301): Interim results of a randomised, double-blind, multicentre, phase 3 trial. Lancet Oncol (2022) 23:209–19. doi: 10.1016/S1470-2045(21)00630-6 35038429

[93] Welsh JW Heymach JV Guo C Menon H Klein K Cushman TR. Phase 1/2 trial of pembrolizumab and concurrent chemoradiation therapy for limited-stage SCLC. J Thorac Oncol (2020) 15:1919–27. doi: 10.1016/j.jtho.2020.08.022 PMC10600713 32916308

[94] Welsh JW Heymach JV Chen D Verma V Cushman TR Hess KR. Phase I trial of pembrolizumab and radiation therapy after induction chemotherapy for extensive-stage small cell lung cancer. J Thorac Oncol (2020) 15:266–73. doi: 10.1016/j.jtho.2019.10.001 31605794

[95] Peters S Pujol JL Dafni U Dómine M Popat S Reck M. Consolidation nivolumab and ipilimumab versus observation in limited-disease small-cell lung cancer after chemo-radiotherapy - results from the randomised phase II ETOP/IFCT 4-12 STIMULI trial. Ann Oncol (2022) 33:67–79. doi: 10.1016/j.annonc.2021.09.011 34562610

[96] Theelen W Peulen H Lalezari F van der Noort V de Vries JF Aerts J. Effect of pembrolizumab after stereotactic body radiotherapy vs pembrolizumab alone on tumor response in patients with advanced non-small cell lung cancer: Results of the PEMBRO-RT phase 2 randomized clinical trial. JAMA Oncol (2019) 5:1276–82. doi: 10.1001/jamaoncol.2019.1478 PMC6624814 31294749

[97] Altorki NK McGraw TE Borczuk AC Saxena A Port JL Stiles BM. Neoadjuvant durvalumab with or without stereotactic body radiotherapy in patients with early-stage non-small-cell lung cancer: a single-centre, randomised phase 2 trial. Lancet Oncol (2021) 22:824–35. doi: 10.1016/S1470-2045(21)00149-2 34015311

[98] Pakkala S Higgins K Chen Z Sica G Steuer C Zhang C. Durvalumab and tremelimumab with or without stereotactic body radiation therapy in relapsed small cell lung cancer: A randomized phase II study. J Immunother Cancer (2020) 8. doi: 10.1136/jitc-2020-001302 PMC7754662 33428583

[99] Bahig H Tonneau M Blais N Wong P Filion E Campeau MP. Stereotactic ablative radiotherapy for oligo-progressive disease refractory to systemic therapy in non-small cell lung cancer: A registry-based phase II randomized trial (SUPPRESS-NSCLC). Clin Transl Radiat Oncol (2022) 33:115–9. doi: 10.1016/j.ctro.2021.12.008 PMC8881202 35243022

[100] Belluomini L Dionisi V Palmerio S Vincenzi S Avancini A Casali M. Study design and rationale for espera trial: A multicentre, randomized, phase II clinical trial evaluating the potential efficacy of adding SBRT to pembrolizumab-pemetrexed maintenance in responsive or stable advanced non-squamous NSCLC after chemo-immunotherapy induction. Clin Lung Cancer (2022) 23:e269–269e272. doi: 10.1016/j.cllc.2021.07.004 34470722

[101] Schild SE Wang X Bestvina CM Williams T Masters G Singh AK. Alliance A082002 -a randomized phase II/III trial of modern immunotherapy-based systemic therapy with or without SBRT for PD-L1-negative, advanced non-small cell lung cancer. Clin Lung Cancer (2022) 23:e317–317e320. doi: 10.1016/j.cllc.2022.04.004 35613998 PMC9634857

