# Supplementary material for: Research landscape and trends of lung cancer radiotherapy: A bibliometric analysis
Source: Front Oncol. 2022 Nov 10;12:1066557. doi: 10.3389/fonc.2022.1066557 (PMC9685815; doi:10.3389/fonc.2022.1066557)
Supplement: Supplementary Material S1 — Detailed search strategy for the papers on lung cancer radiotherapy. [file DataSheet_1.docx]

**Search Strategy**

TI= (((lung OR pulmonary) NEAR/0 (cancer OR carcinoma OR neoplasm OR adenocarcinoma)) OR (NSCLC) OR (SCLC)) AND (TI=((radiotherapy) OR (radiation) OR (irradiation) OR (chemoradiotherapy) OR (chemoradiation) OR (SBRT) OR (SABR)) OR AB=((radiotherapy) OR (radiation) OR (irradiation) OR (chemoradiotherapy) OR (chemoradiation) OR (SBRT) OR (SABR))) AND DT=(Article) NOT DT=((Book Chapter) OR (Abstract) OR (Meeting) OR (Review) OR (Case Report) OR (Editorial Material) OR (Correction) OR (Letter) OR (Reference Material) OR (Retracted Publication) OR (Data Paper) OR (News) OR (Expression Of Concern) OR (Retraction) OR (Report)) NOT TI=((guideline) OR (consensus recommendations) OR (meta-analyses) OR (meta-analysis) OR (meta analysis) OR (data pooling) OR (pooled) OR (overview) OR (current status) OR (development) OR (review) OR (progress to date) OR (interstitial lung disease) OR (case) OR (cases))

**Database**: Web of Science (Science Citation Indexing Expanded database)

**Publication time**: 2000–2022

**Design principles**

1. The publications should be related to lung cancer radiotherapy. Therefore, the search strategy should include “lung cancer” and “radiotherapy”.
2. The search strategy should include the synonyms of “lung cancer” and “radiotherapy” to avoid omission.
3. The publication titles should contain synonyms of “lung cancer”. Because some papers regarding other diseases also contain “lung cancer” in abstract or keywords.
4. The publication titles or abstracts should contain synonyms of “radiotherapy”. Because some papers related to radiotherapy do not contain “radiotherapy” in their titles, and some irrelevant papers contain “radiotherapy” only in keywords.
5. The document type should be “Article”.
