# Supplementary material for: Research landscape and trends of lung cancer radiotherapy: A bibliometric analysis
Source: Front Oncol. 2022 Nov 10;12:1066557. doi: 10.3389/fonc.2022.1066557 (PMC9685815; doi:10.3389/fonc.2022.1066557)
Supplement: Supplementary Table S1 — The 100 top-papers on lung cancer radiotherapy. [file Table_1.docx]

| **TABLE S1** The 100 most cited papers in lung cancer radiotherapy from 2000 to 2022. | | | | | | | |
| --- | --- | --- | --- | --- | --- | --- | --- |
| Rank | Title | Corresponding author | Journal | Year | Total citation | Average citation per year (rank) |  |
| 1 | Durvalumab after Chemoradiotherapy in Stage III Non-Small-Cell Lung Cancer | Antonia SJ | N. Engl. J. Med. | 2017 | 2110 | 436.55 (1) |  |
| 2 | Stereotactic Body Radiation Therapy for Inoperable Early Stage Lung Cancer | Timmerman R | JAMA-J. Am. Med. Assoc. | 2010 | 1805 | 144.4 (6) |  |
| 3 | Cisplatin-based adjuvant chemotherapy in patients with completely resected non-small-cell lung cancer | LeChevalier T | N. Engl. J. Med. | 2004 | 1732 | 92.79 (13) |  |
| 4 | Overall Survival with Durvalumab after Chemoradiotherapy in Stage III NSCLC | Antonia SJ | N. Engl. J. Med. | 2018 | 1358 | 362.13 (2) |  |
| 5 | Standard-dose versus high-dose conformal radiotherapy with concurrent and consolidation carboplatin plus paclitaxel with or without cetuximab for patients with stage IIIA or IIIB non-small-cell lung cancer (RTOG 0617): a randomised, two-by-two factorial phase 3 study | Bradley JD | Lancet Oncol. | 2015 | 1240 | 163.52 (4) |  |
| 6 | Adjuvant vinorelbine plus cisplatin versus observation in patients with completely resected stage IB-IIIA non-small-cell lung cancer (Adjuvant Navelbine International Trialist Association [ANITA]): a randomised controlled trial | Douillard JY | Lancet Oncol. | 2006 | 1153 | 72.06 (18) |  |
| 7 | Excessive toxicity when treating central tumors in a phase II study of stereotactic body radiation therapy for medically inoperable early-stage lung cancer | Timmerman R | J. Clin. Oncol. | 2006 | 1074 | 67.48 (23) |  |
| 8 | Radiotherapy plus chemotherapy with or without surgical resection for stage III non-small-cell lung cancer: a phase III randomised controlled trial | Albain KS | Lancet | 2009 | 993 | 75.9 (15) |  |
| 9 | Akt/protein kinase B is constitutively active in non-small cell lung cancer cells and promotes cellular survival and resistance to chemotherapy and radiation | Dennis PA | Cancer Res. | 2001 | 812 | 38.06 (62) |  |
| 10 | Lung Cancer: Epidemiology, Etiology, and Prevention | DelaCruz CS | Clin. Chest Med. | 2011 | 802 | 74.6 (16) |  |
| 11 | Sequential vs Concurrent Chemoradiation for Stage III Non-Small Cell Lung Cancer: Randomized Phase III Trial RTOG 9410 | Curran WJ | J. Natl. Cancer Inst. | 2011 | 785 | 71.91 (19) |  |
| 12 | Long-term mortality from heart disease and lung cancer after radiotherapy for early breast cancer: prospective cohort study of about 300 000 women in US SEER cancer registries | Darby SC | Lancet Oncol. | 2005 | 739 | 43.26 (50) |  |
| 13 | Prophylactic cranial irradiation in extensive small-cell lung cancer | Slotman B | N. Engl. J. Med. | 2007 | 735 | 48.73 (39) |  |
| 14 | Hypofractionated stereotactic radiotherapy (HypoFXSRT) for stage I non-small cell lung cancer: Updated results of 257 patients in a Japanese multi-institutional study | Onishi H | J. Thorac. Oncol. | 2007 | 707 | 46.62 (47) |  |
| 15 | Adjuvant Paclitaxel Plus Carboplatin Compared With Observation in Stage IB Non-Small-Cell Lung Cancer: CALGB 9633 With the Cancer and Leukemia Group B, Radiation Therapy Oncology Group, and North Central Cancer Treatment Group Study Groups | Strauss GM | J. Clin. Oncol. | 2008 | 668 | 48.29 (41) |  |
| 16 | Stereotactic hypofractionated high-dose irradiation for stage I nonsmall cell lung carcinoma - Clinical outcomes in 245 subjects in a Japanese multinstitutional study | Onishi H | Cancer | 2004 | 653 | 36.45 (65) |  |
| 17 | Outcome in a Prospective Phase II Trial of Medically Inoperable Stage I Non-Small-Cell Lung Cancer Patients Treated With Stereotactic Body Radiotherapy | Baumann P | J. Clin. Oncol. | 2009 | 649 | 49.29 (37) |  |
| 18 | Small-cell lung cancer | vanMeerbeeck JP | Lancet | 2011 | 648 | 59.82 (28) |  |
| 19 | Durvalumab plus platinum-etoposide versus platinum-etoposide in first-line treatment of extensive-stage small-cell lung cancer (CASPIAN): a randomised, controlled, open-label, phase 3 trial | Paz-Ares L | Lancet | 2019 | 612 | 216 (3) |  |
| 20 | Local consolidative therapy versus maintenance therapy or observation for patients with oligometastatic non-small-cell lung cancer without progression after first-line systemic therapy: a multicentre, randomised, controlled, phase 2 study | Gomez DR | Lancet Oncol. | 2016 | 611 | 106.26 (9) |  |
| 21 | STEREOTACTIC BODY RADIATION THERAPY FOR EARLY-STAGE NON-SMALL-CELL LUNG CARCINOMA: FOUR-YEAR RESULTS OF A PROSPECTIVE PHASE II STUDY | Fakiris AJ | Int. J. Radiat. Oncol. Biol. Phys. | 2009 | 605 | 47.14 (45) |  |
| 22 | Previous radiotherapy and the clinical activity and toxicity of pembrolizumab in the treatment of non-small-cell lung cancer: a secondary analysis of the KEYNOTE-001 phase 1 trial | Lee P | Lancet Oncol. | 2017 | 580 | 112.26 (8) |  |
| 23 | Non-small-cell lung cancer | Goldstraw P | Lancet | 2011 | 532 | 49.11 (38) |  |
| 24 | Screening for lung cancer with low-dose spiral computed tomography | Swensen SJ | Am. J. Respir. Crit. Care Med. | 2002 | 524 | 25.46 (114) |  |
| 25 | Extracranial stereotactic radioablation - Results of a phase I study in medically inoperable stage I non-small cell lung cancer | Timmerman R | Chest | 2003 | 522 | 27.72 (99) |  |
| 26 | Zoledronic acid versus placebo in the treatment of skeletal metastases in patients with lung cancer and other solid tumors: A phase III, double-blind, randomized trial - The zoledronic acid lung cancer and other solid tumors study group | Rosen LS | J. Clin. Oncol. | 2003 | 520 | 27.25 (100) |  |
| 27 | Comparison of different methods for delineation of F-18-FDG PET-positive tissue for target volume definition in radiotherapy of patients with non-small cell lung cancer | Nestle U | J. Nucl. Med. | 2005 | 515 | 30.15 (85) |  |
| 28 | Clinical outcomes of a phase I/II study of 48 Gy of stereotactic body radiotherapy in 4 fractions for primary lung cancer using a stereotactic body frame | Nagata Y | Int. J. Radiat. Oncol. Biol. Phys. | 2005 | 498 | 29.73 (89) |  |
| 29 | Local Consolidative Therapy Vs. Maintenance Therapy or Observation for Patients With Oligometastatic Non-Small-Cell Lung Cancer: Long-Term Results of a Multi-Institutional, Phase II, Randomized Study | Gomez DR | J. Clin. Oncol. | 2019 | 495 | 152.31 (5) |  |
| 30 | Randomized controlled trial of resection versus radiotherapy after induction chemotherapy in stage IIIA-N2 non-small-cell lung cancer | vanMeerbeeck JP | JNCI-J. Natl. Cancer Inst. | 2007 | 492 | 31.74 (76) |  |
| 31 | Preoperative chemotherapy followed by surgery compared with primary surgery in resectable stage I (except T1N0), II, and IIIa non-small-cell lung cancer | Depierre A | J. Clin. Oncol. | 2002 | 492 | 23.81 (122) |  |
| 32 | An Abscopal Response to Radiation and Ipilimumab in a Patient with Metastatic Non-Small Cell Lung Cancer | Formenti SC | Cancer Immunol. Res. | 2013 | 490 | 56 (30) |  |
| 33 | Molecular predictors of response to epidermal growth factor receptor antagonists in non-small-cell lung cancer | Haber DA | J. Clin. Oncol. | 2007 | 490 | 31.44 (78) |  |
| 34 | Long-term efficacy and safety of zoledronic acid in the treatment of skeletal metastases in patients with nonsmall cell lung carcinoma and other solid tumors - A randomized, phase III, double-blind, placebo-controlled trial | Rosen LS | Cancer | 2004 | 484 | 26.52 (104) |  |
| 35 | Final results of phase III trial in regionally advanced unresectable non-small cell lung cancer - Radiation Therapy Oncology Group, Eastern Cooperative Oncology Group, and Southwest Oncology Group | Sause W | Chest | 2000 | 478 | 21.17 (151) |  |
| 36 | Consolidative Radiotherapy for Limited Metastatic Non-Small-Cell Lung Cancer A Phase 2 Randomized Clinical Trial | Iyengar P | JAMA Oncol. | 2018 | 470 | 100.71 (11) |  |
| 37 | The Novel Histologic International Association for the Study of Lung Cancer/American Thoracic Society/European Respiratory Society Classification System of Lung Adenocarcinoma Is a Stage-Independent Predictor of Survival | Warth A | J. Clin. Oncol. | 2012 | 468 | 45.29 (49) |  |
| 38 | Local Ablative Therapy of Oligoprogressive Disease Prolongs Disease Control by Tyrosine Kinase Inhibitors in Oncogene-Addicted Non-Small-Cell Lung Cancer | Weickhardt AJ | J. Thorac. Oncol. | 2012 | 459 | 47.08 (46) |  |
| 39 | Alectinib in Crizotinib-Refractory ALK-Rearranged Non-Small-Cell Lung Cancer: A Phase II Global Study | Ou SHI | J. Clin. Oncol. | 2016 | 457 | 70.31 (21) |  |
| 40 | Randomized study of adjuvant chemotherapy for completely resected stage I, II, or IIIA non-small-cell lung cancer | Scagliotti GV | JNCI-J. Natl. Cancer Inst. | 2003 | 447 | 23.63 (125) |  |
| 41 | Phase III trial of maintenance gefitinib or placebo after concurrent chemoradiotherapy and docetaxel consolidation in inoperable stage III non-small-cell lung cancer: SWOG S0023 | Kelly K | J. Clin. Oncol. | 2008 | 442 | 30.84 (82) |  |
| 42 | Outcomes of risk-adapted fractionated stereotactic radiotherapy for stage I non-small-cell lung cancer | Lagerwaard FJ | Int. J. Radiat. Oncol. Biol. Phys. | 2008 | 431 | 29.72 (90) |  |
| 43 | Clinical Experience With Crizotinib in Patients With Advanced ALK-Rearranged Non-Small-Cell Lung Cancer and Brain Metastases | Costa DB | J. Clin. Oncol. | 2015 | 430 | 59.31 (29) |  |
| 44 | STEREOTACTIC BODY RADIOTHERAPY (SBRT) FOR OPERABLE STAGE I NON SMALL-CELL LUNG CANCER: CAN SBRT BE COMPARABLE TO SURGERY? | Onishi H | Int. J. Radiat. Oncol. Biol. Phys. | 2011 | 428 | 39.81 (54) |  |
| 45 | Phase III study of concurrent versus sequential thoracic radiotherapy in combination with cisplatin and etoposide for limited-stage small-cell lung cancer: Results of the Japan Clinical Oncology Group Study 9104 | Saijo N | J. Clin. Oncol. | 2002 | 425 | 21.07 (152) |  |
| 46 | Randomized phase III trial of sequential chemoradiotherapy compared with concurrent chemoradiotherapy in locally advanced non-small-cell lung cancer: Groupe Lyon-Saint-Etienne d'Oncologie Thoracique-Groupe Francais de Pneumo-Cancerologie NPC 95-01 Study | Fournel P | J. Clin. Oncol. | 2005 | 406 | 23.88 (121) |  |
| 47 | Impact of FDG-PET on radiation therapy volume delineation in non-small-cell lung cancer | Bradley J | Int. J. Radiat. Oncol. Biol. Phys. | 2004 | 405 | 22.09 (138) |  |
| 48 | Lung cancer in elderly patients: An analysis of the surveillance, epidemiology, and end results database | Ramalingam SS | J. Clin. Oncol. | 2007 | 400 | 27.12 (101) |  |
| 49 | Lung cancer following chemotherapy and radiotherapy for Hodgkin's disease | Travis LB | JNCI-J. Natl. Cancer Inst. | 2002 | 398 | 19.34 (179) |  |
| 50 | Impact of Intensity-Modulated Radiation Therapy Technique for Locally Advanced Non-Small-Cell Lung Cancer: A Secondary Analysis of the NRG Oncology RTOG 0617 Randomized Clinical Trial | Chun SG | J. Clin. Oncol. | 2017 | 388 | 68.47 (22) |  |
| 51 | Stereotactic body radiation therapy of early-stage non-small-cell lung carcinoma: Phase I study | McGarry RC | Int. J. Radiat. Oncol. Biol. Phys. | 2005 | 384 | 22.81 (131) |  |
| 52 | High-dose radiation improved local tumor control and overall survival in patients with inoperable/unresectable non-small-cell lung cancer: Long-term results of a radiation dose escalation study | Kong FM | Int. J. Radiat. Oncol. Biol. Phys. | 2005 | 382 | 22.58 (134) |  |
| 53 | Radiotherapy induces responses of lung cancer to CTLA-4 blockade | Formenti SC;Demaria S | Nat. Med. | 2018 | 379 | 101.07 (10) |  |
| 54 | Dexamethasone and supportive care with or without whole brain radiotherapy in treating patients with non-small cell lung cancer with brain metastases unsuitable for resection or stereotactic radiotherapy (QUARTZ): results from a phase 3, non-inferiority, randomised trial | Langley RE | Lancet | 2016 | 370 | 62.54 (27) |  |
| 55 | Radiation-induced pulmonary toxicity: A dose-volume histogram analysis in 201 patients with lung cancer | Marks LB | Int. J. Radiat. Oncol. Biol. Phys. | 2001 | 369 | 17.71 (200) |  |
| 56 | Computed tomography-guided frameless stereotactic radiotherapy for Stage I non-small-cell lung cancer: A 5-year experience | Uematsu M | Int. J. Radiat. Oncol. Biol. Phys. | 2001 | 363 | 17.42 (205) |  |
| 57 | Phase III Study of Cisplatin, Etoposide, and Concurrent Chest Radiation With or Without Consolidation Docetaxel in Patients With Inoperable Stage III Non-Small-Cell Lung Cancer: The Hoosier Oncology Group and US Oncology | Hanna N | J. Clin. Oncol. | 2008 | 363 | 26.4 (105) |  |
| 58 | Oct-4 Expression Maintained Cancer Stem-Like Properties in Lung Cancer-Derived CD133-Positive Cells | Ku HH | PLoS One | 2008 | 362 | 25.55 (112) |  |
| 59 | Positron emission tomography is superior to computed tomography scanning for response-assessment after radical radiotherapy or chemoradiotherapy in patients with non-small-cell lung cancer | MacManus MP | J. Clin. Oncol. | 2003 | 351 | 18.08 (194) |  |
| 60 | Impact of Introducing Stereotactic Lung Radiotherapy for Elderly Patients With Stage I Non-Small-Cell Lung Cancer: A Population-Based Time-Trend Analysis | Palma D | J. Clin. Oncol. | 2010 | 342 | 29.11 (95) |  |
| 61 | Effect of Pembrolizumab After Stereotactic Body Radiotherapy vs Pembrolizumab Alone on Tumor Response in Patients With Advanced Non-Small Cell Lung Cancer: Results of the PEMBRO-RT Phase 2 Randomized Clinical Trial | Theelen WSME | JAMA Oncol. | 2019 | 342 | 114 (7) |  |
| 62 | Postoperative radiotherapy for stage II or III non-small-cell lung cancer using th surveillance, epidemiology, and end results database | Lally BE | J. Clin. Oncol. | 2006 | 340 | 21.03 (154) |  |
| 63 | Tecemotide (L-BLP25) versus placebo after chemoradiotherapy for stage III non-small-cell lung cancer (START): a randomised, double-blind, phase 3 trial | Butts C | Lancet Oncol. | 2014 | 339 | 39.12 (58) |  |
| 64 | Prognostic impact of hypoxia imaging with F-18-misonidazole PET in non-small cell lung cancer and head and neck cancer before radiotherapy | Eschmann SM | J. Nucl. Med. | 2005 | 337 | 19.17 (181) |  |
| 65 | Induction chemotherapy followed by chemoradiotherapy compared with chemoradiotherapy alone for regionally advanced unresectable stage III non-small-cell lung cancer: Cancer and Leukemia Group B | Vokes EE | J. Clin. Oncol. | 2007 | 335 | 21.85 (142) |  |
| 66 | A randomized trial of postoperative adjuvant therapy in patients with completely resected stage II or IIIa non-small-cell lung cancer. | Keller SM | N. Engl. J. Med. | 2000 | 334 | 15.24 (262) |  |
| 67 | Benefits and Harms of Computed Tomography Lung Cancer Screening Strategies: A Comparative Modeling Study for the US Preventive Services Task Force | deKoning HJ | Ann. Intern. Med. | 2014 | 334 | 39.29 (57) |  |
| 68 | Cisplatin and etoposide regimen is superior to cyclophosphamide, epirubicin, and vincristine regimen in small-cell lung cancer: Results from a randomized phase III trial with 5 years' follow-up | Bremnes RM | J. Clin. Oncol. | 2002 | 332 | 16.81 (220) |  |
| 69 | Concurrent versus sequential chemoradiotherapy with cisplatin and vinorelbine in locally advanced non-small cell lung cancer: a randomized study | Zatloukal P | Lung Cancer | 2004 | 331 | 18.47 (189) |  |
| 70 | Patterns of disease recurrence after stereotactic ablative radiotherapy for early stage non-small-cell lung cancer: a retrospective analysis | Lagerwaard FJ | Lancet Oncol. | 2012 | 323 | 32.03 (75) |  |
| 71 | Radiation risks potentially associated with low-dose CT screening of adult smokers for lung cancer | Brenner DJ | Radiology | 2004 | 322 | 17.56 (202) |  |
| 72 | Outcomes After Stereotactic Lung Radiotherapy or Wedge Resection for Stage I Non-Small-Cell Lung Cancer | Grills IS | J. Clin. Oncol. | 2010 | 321 | 25.51 (113) |  |
| 73 | Toxicity and outcome results of RTOG 9311: A phase I-II dose-escalation study using three-dimensional conformal radiotherapy in patients with inoperable non-small-cell lung carcinoma | Bradley J | Int. J. Radiat. Oncol. Biol. Phys. | 2005 | 319 | 18.14 (193) |  |
| 74 | Combined chemoradiotherapy regimens of paclitaxel and carboplatin for locally advanced non-small-cell lung cancer: A randomized phase II locally advanced multi-modality protocol | Belani CP | J. Clin. Oncol. | 2005 | 317 | 18.65 (186) |  |
| 75 | Lung cancer: Diagnosis and management | Collins LG | Am. Fam. Physician | 2007 | 311 | 19.85 (172) |  |
| 76 | Assessing the relationship between lung cancer risk and emphysema detected on low-dose CT of the chest | Zulueta JJ | Chest | 2007 | 307 | 20.81 (159) |  |
| 77 | Are Pretreatment F-18-FDG PET Tumor Textural Features in Non-Small Cell Lung Cancer Associated with Response and Survival After Chemoradiotherapy? | Cook GJR | J. Nucl. Med. | 2013 | 306 | 31.66 (77) |  |
| 78 | Evaluation of microscopic tumor extension in non-small-cell lung cancer for three-dimensional conformal radiotherapy planning | Giraud P | Int. J. Radiat. Oncol. Biol. Phys. | 2000 | 302 | 13.83 (314) |  |
| 79 | Use of thoracic radiotherapy for extensive stage small-cell lung cancer: a phase 3 randomised controlled trial | Slotman BJ | Lancet | 2015 | 293 | 38.22 (60) |  |
| 80 | The deep inspiration breath-hold technique in the treatment of inoperable non-small-cell lung cancer | Rosenzweig KE | Int. J. Radiat. Oncol. Biol. Phys. | 2000 | 291 | 13.18 (339) |  |
| 81 | Phase II Trial of Erlotinib Plus Concurrent Whole-Brain Radiation Therapy for Patients With Brain Metastases From Non-Small-Cell Lung Cancer | Welsh JW | J. Clin. Oncol. | 2013 | 286 | 30.11 (87) |  |
| 82 | Lactate dehydrogenase-5 (LDH-5) overexpression in non-small-cell lung cancer tissues is linked to tumour hypoxia, angiogenic factor production and poor prognosis | Koukourakis MI | Br. J. Cancer | 2003 | 286 | 15.05 (267) |  |
| 83 | Randomized phase II study of cisplatin with gemcitabine or paclitaxel or vinorelbine as induction chemotherapy followed by concomitant chemoradiotherapy for stage IIIB non-small-cell lung cancer: Cancer and leukemia group B study 9431 | Vokes EE | J. Clin. Oncol. | 2002 | 283 | 14.21 (300) |  |
| 84 | Chemotherapy for patients with non-small cell lung cancer: the surgical setting of the Big Lung Trial | Stephens RJ | Eur. J. Cardio-Thorac. Surg. | 2004 | 282 | 15.52 (255) |  |
| 85 | Stereotactic radiotherapy for primary lung cancer and pulmonary metastases: A noninvasive treatment approach in medically inoperable patients | Wulf J | Int. J. Radiat. Oncol. Biol. Phys. | 2004 | 276 | 15.33 (260) |  |
| 86 | Induction chemoradiation and surgical resection for superior sulcus non-small-cell lung carcinomas: Long-term results of Southwest Oncology Group trial 9416 (Intergroup trial 0160) | Rusch VW | J. Clin. Oncol. | 2007 | 274 | 17.49 (203) |  |
| 87 | Analysis of clinical and dosimetric factors associated with treatment-related pneumonitis (TRP) in patients with non-small-cell lung cancer (NSCLC) treated with concurrent chemotherapy and three-dimensional conformal radiotherapy (3D-CRT) | Liao ZX | Int. J. Radiat. Oncol. Biol. Phys. | 2006 | 273 | 17.33 (207) |  |
| 88 | An Individual Patient Data Metaanalysis of Outcomes and Prognostic Factors After Treatment of Oligometastatic Non-Small-Cell Lung Cancer | Rodrigues GB | Clin. Lung Cancer | 2014 | 272 | 34 (70) |  |
| 89 | Gefitinib in patients with brain metastases from non-small-cell lung cancer: a prospective trial | Ceresoli GL | Ann. Oncol. | 2004 | 265 | 14.59 (288) |  |
| 90 | Prophylactic cranial irradiation versus observation in patients with extensive-disease small-cell lung cancer: a multicentre, randomised, open-label, phase 3 trial | Yamamoto N | Lancet Oncol. | 2017 | 263 | 49.31 (36) |  |
| 91 | Outcomes of Stereotactic Ablative Radiotherapy in Patients With Potentially Operable Stage I Non-Small Cell Lung Cancer | Lagerwaard FJ | Int. J. Radiat. Oncol. Biol. Phys. | 2012 | 257 | 24.87 (116) |  |
| 92 | PROCLAIM: Randomized Phase III Trial of Pemetrexed-Cisplatin or Etoposide-Cisplatin Plus Thoracic Radiation Therapy Followed by Consolidation Chemotherapy in Locally Advanced Nonsquamous Non-Small-Cell Lung Cancer | Senan S | J. Clin. Oncol. | 2016 | 257 | 39.54 (56) |  |
| 93 | Impact of postoperative radiation therapy on survival in patients with complete resection and Stage I, II, or IIIA non-small-cell lung cancer treated with adjuvant chemotherapy: The Adjuvant Navelbine International Trialist Association (ANITA) randomized trial | Douillard JY | Int. J. Radiat. Oncol. Biol. Phys. | 2008 | 256 | 18.51 (188) |  |
| 94 | Prospective Trial of Stereotactic Body Radiation Therapy for Both Operable and Inoperable T1N0M0 Non-Small Cell Lung Cancer: Japan Clinical Oncology Group Study JCOG0403 | Nagata Y | Int. J. Radiat. Oncol. Biol. Phys. | 2015 | 255 | 37.78 (63) |  |
| 95 | Radiotherapy treatment planning for patients with non-small cell lung cancer using positron emission tomography (PET) | Erdi YE | Radiother. Oncol. | 2002 | 255 | 12.34 (362) |  |
| 96 | Concurrent cisplatin, etoposide, and chest radiotherapy in Pathologic stage IIIB non-small-cell lung cancer: A Southwest Oncology Group Phase II Study, SWOG 9019 | Albain KS | J. Clin. Oncol. | 2002 | 255 | 12.7 (354) |  |
| 97 | Escape from therapy-induced accelerated cellular senescence in p53-null lung cancer cells and in human lung cancers | Wu DY | Cancer Res. | 2005 | 254 | 14.58 (289) |  |
| 98 | Concurrent once-daily versus twice-daily chemoradiotherapy in patients with limited-stage small-cell lung cancer (CONVERT): an open-label, phase 3, randomised, superiority trial | Faivre-Finn C | Lancet Oncol. | 2017 | 253 | 49.77 (35) |  |
| 99 | Phase III Trial of Prophylactic Cranial Irradiation Compared With Observation in Patients With Locally Advanced Non-Small-Cell Lung Cancer: Neurocognitive and Quality-of-Life Analysis | Sun A | J. Clin. Oncol. | 2011 | 252 | 21.6 (145) |  |
| 100 | Gemcitabine plus best supportive care (BSC) vs BSC in inoperable non-small cell lung cancer - a randomized trial with quality of life as the primary outcome | Anderson H | Br. J. Cancer | 2000 | 252 | 11.41 (406) |  |
