# Supplementary material for: Research landscape and trends of lung cancer radiotherapy: A bibliometric analysis
Source: Front Oncol. 2022 Nov 10;12:1066557. doi: 10.3389/fonc.2022.1066557 (PMC9685815; doi:10.3389/fonc.2022.1066557)
Supplement: Supplementary Table S2 — The journals with top-papers on lung cancer radiotherapy. [file Table_2.docx]

| **TABLE S2** \| The journals with top-cited papers in lung cancer radiotherapy from 2000 to 2022. | | | |
| --- | --- | --- | --- |
| **Journals with top-cited papers** | **Top-Cited Paper number** | **Paper number (2000-2019)** | **TPR** |
| J. Clin. Oncol. | 29 | 150 | 19.33% |
| Int. J. Radiat. Oncol. Biol. Phys. | 17 | 634 | 2.68% |
| Lancet Oncol. | 9 | 20 | 45.00% |
| Lancet | 6 | 7 | 85.71% |
| N. Engl. J. Med. | 5 | 5 | 100.00% |
| Chest | 3 | 54 | 5.56% |
| J. Nucl. Med. | 3 | 32 | 9.38% |
| JNCI-J. Natl. Cancer Inst. | 3 | 13 | 23.08% |
| Br. J. Cancer | 2 | 52 | 3.85% |
| Cancer | 2 | 117 | 1.71% |
| Cancer Res. | 2 | 27 | 7.41% |
| J. Thorac. Oncol. | 2 | 334 | 0.60% |
| JAMA Oncol. | 2 | 13 | 15.38% |
| Am. Fam. Physician | 1 | 2 | 50.00% |
| Am. J. Respir. Crit. Care Med. | 1 | 4 | 25.00% |
| Ann. Intern. Med. | 1 | 1 | 100.00% |
| Ann. Oncol. | 1 | 64 | 1.56% |
| Cancer Immunol. Res. | 1 | 2 | 50.00% |
| Clin. Chest Med. | 1 | 9 | 11.11% |
| Clin. Lung Cancer | 1 | 204 | 0.49% |
| Eur. J. Cardio-Thorac. Surg. | 1 | 67 | 1.49% |
| J. Natl. Cancer Inst. | 1 | 2 | 50.00% |
| JAMA-J. Am. Med. Assoc. | 1 | 1 | 100.00% |
| Lung Cancer | 1 | 527 | 0.19% |
| Nat. Med. | 1 | 1 | 100.00% |
| PLoS One | 1 | 87 | 1.15% |
| Radiology | 1 | 14 | 7.14% |
| Radiother. Oncol. | 1 | 370 | 0.27% |
