# Supplementary material for: Research landscape and trends of lung cancer radiotherapy: A bibliometric analysis
Source: Front Oncol. 2022 Nov 10;12:1066557. doi: 10.3389/fonc.2022.1066557 (PMC9685815; doi:10.3389/fonc.2022.1066557)
Supplement: Supplementary Table S3 — The papers on lung cancer radiotherapy published in major journals since 2020. [file Table_3.docx]

| **TABLE S3** The 110 papers in lung cancer radiotherapy published in top-journals since 2020. | | | | | |
| --- | --- | --- | --- | --- | --- |
| No. | Title | Corresponding author | Journal | Year | Total citation |
| 1 | Exosomal protein angiopoietin-like 4 mediated radioresistance of lung cancer by inhibiting ferroptosis under hypoxic microenvironment | Shao CL; Zhang JH | Br. J. Cancer | 2022 | 0 |
| 2 | Phase I Study of Accelerated Hypofractionated Proton Therapy and Chemotherapy for Locally Advanced Non-Small Cell Lung Cancer | Robinson C | Int. J. Radiat. Oncol. Biol. Phys. | 2022 | 0 |
| 3 | Phase I Study of Accelerated Hypofractionated Proton Therapy and Chemotherapy for Locally Advanced Non-Small Cell Lung Cancer | Robinson C | Int. J. Radiat. Oncol. Biol. Phys. | 2022 | 0 |
| 4 | Chemoradiation with Hypofractionated Proton Therapy in Stage II-III Non-Small Cell Lung Cancer: A Proton Collaborative Group Phase 2 Trial | Hoppe BS | Int. J. Radiat. Oncol. Biol. Phys. | 2022 | 0 |
| 5 | Prognostic and Predictive Role of PD-L1 Expression in Stage Ill Non-small Cell Lung Cancer Treated With Definitive Chemoradiation and Adjuvant Durvalumab | Green MD | Int. J. Radiat. Oncol. Biol. Phys. | 2022 | 0 |
| 6 | Chemoradiation with Hypofractionated Proton Therapy in Stage II-III Non-Small Cell Lung Cancer: A Proton Collaborative Group Phase 2 Trial | Hoppe BS | Int. J. Radiat. Oncol. Biol. Phys. | 2022 | 2 |
| 7 | CLINICAL INVESTIGATION Prognostic and Predictive Role of PD-L1 Expression in Stage III Non-small Cell Lung Cancer Treated With Definitive Chemoradiation and Adjuvant Durvalumab | Green MD | Int. J. Radiat. Oncol. Biol. Phys. | 2022 | 0 |
| 8 | Risk of Cardiovascular Toxicity According to Tumor Laterality Among Older Patients With Early Stage Non-small Cell Lung Cancer Treated With Radiation Therapy | Sigel K | Chest | 2022 | 0 |
| 9 | Effects of Tumor Mutational Burden and Gene Alterations Associated with Radiation Response on Outcomes of Postoperative Radiation Therapy in Non-Small Cell Lung Cancer | Shaverdian N | Int. J. Radiat. Oncol. Biol. Phys. | 2022 | 0 |
| 10 | Dynamics of Circulating Immune Cells During Chemoradiotherapy in Patients with Non-Small Cell Lung Cancer Support Earlier Administration of Anti-PD-1/PD-L1 | Ahn MJ | Int. J. Radiat. Oncol. Biol. Phys. | 2022 | 2 |
| 11 | Timing of Adjuvant Durvalumab Initiation Is Not Associated With Outcomes in Stage III Non-small Cell Lung Cancer | Green MD | Int. J. Radiat. Oncol. Biol. Phys. | 2022 | 3 |
| 12 | Lung Cancer: Diagnosis, Treatment Principles, and Screening | Kim J | Am. Fam. Physician | 2022 | 0 |
| 13 | Five-Year Survival Outcomes From the PACIFIC Trial: Durvalumab After Chemoradiotherapy in Stage III Non-Small-Cell Lung Cancer | Spigel DR | J. Clin. Oncol. | 2022 | 36 |
| 14 | First-in-Humans Evaluation of a PD-L1-Binding Peptide PET Radiotracer in Non-Small Cell Lung Cancer Patients | Zhu H; Yang Z; Li N | J. Nucl. Med. | 2022 | 13 |
| 15 | Impact of Local Recurrence on Cause-Specific Death After Stereotactic Body Radiotherapy for Early-Stage Non-Small Cell Lung Cancer: Dynamic Prediction Using Landmark Model | Matsuo Y | Int. J. Radiat. Oncol. Biol. Phys. | 2022 | 0 |
| 16 | Comprehensive Next-Generation Sequencing Reveals Novel Predictive Biomarkers of Recurrence and Thoracic Toxicity Risks After Chemoradiation Therapy in Limited Stage Small Cell Lung Cancer | Yu JM; Yuan SH | Int. J. Radiat. Oncol. Biol. Phys. | 2022 | 2 |
| 17 | Golgi Phosphoprotein 3 Confers Radioresistance via Stabilizing EGFR in Lung Adenocarcinoma | Han W | Int. J. Radiat. Oncol. Biol. Phys. | 2022 | 0 |
| 18 | Association Between Physician- and Patient-Reported Symptoms in Patients Treated With Definitive Radiation Therapy for Locally Advanced Lung Cancer in a Statewide Consortium | Jolly S | Int. J. Radiat. Oncol. Biol. Phys. | 2022 | 1 |
| 19 | Dosimetric Planning Tradeoffs to Reduce Heart Dose Using Machine Learning-Guided Decision Support Software in Patients with Lung Cancer | Atkins KM | Int. J. Radiat. Oncol. Biol. Phys. | 2022 | 1 |
| 20 | Integration of Deep Learning Radiomics and Counts of Circulating Tumor Cells Improves Prediction of Outcomes of Early Stage NSCLC Patients Treated With Stereotactic Body Radiation Therapy | Fan Y | Int. J. Radiat. Oncol. Biol. Phys. | 2022 | 0 |
| 21 | Surgical Outcomes for Early Stage Non-small Cell Lung Cancer at Facilities With Stereotactic Body Radiation Therapy Programs | Moghanaki D | Chest | 2022 | 1 |
| 22 | Sugemalimab versus placebo after concurrent or sequential chemoradiotherapy in patients with locally advanced, unresectable, stage III non-small-cell lung cancer in China (GEMSTONE-301): interim results of a randomised, double-blind, multicentre, phase 3 trial | Wu YL | Lancet Oncol. | 2022 | 9 |
| 23 | Durvalumab plus tremelimumab alone or in combination with low-dose or hypofractionated radiotherapy in metastatic non-small-cell lung cancer refractory to previous PD(L)-1 therapy: an open-label, multicentre, randomised, phase 2 trial | Schoenfeld JD | Lancet Oncol. | 2022 | 14 |
| 24 | American Radium Society Appropriate Use Criteria for Radiation Therapy in Oligometastatic or Oligoprogressive Non-Small Cell Lung Cancer | Amini A | Int. J. Radiat. Oncol. Biol. Phys. | 2022 | 6 |
| 25 | Radiosensitivity-Specific Proteomic and Signaling Pathway Network of Non-Small Cell Lung Cancer (NSCLC) | Fu XL | Int. J. Radiat. Oncol. Biol. Phys. | 2022 | 0 |
| 26 | Randomized Trial of First-Line Tyrosine Kinase Inhibitor With or Without Radiotherapy for Synchronous Oligometastatic EGFR-Mutated Non-Small Cell Lung Cancer | Zeng M | JNCI-J. Natl. Cancer Inst. | 2022 | 9 |
| 27 | Postoperative radiotherapy versus no postoperative radiotherapy in patients with completely resected non-small-cell lung cancer and proven mediastinal N2 involvement (Lung ART): an open-label, randomised, phase 3 trial | Le Pechoux C | Lancet Oncol. | 2022 | 17 |
| 28 | Secondary Infections After Diagnosis of Severe Radiation Pneumonitis (SRP) Among Patients With Non-Small Cell Lung Cancer: Pathogen Distributions, Choice of Empirical Antibiotics, and the Value of Empirical Antifungal Treatment | Gong YL | Int. J. Radiat. Oncol. Biol. Phys. | 2022 | 1 |
| 29 | Coronary Artery Calcifications and Cardiac Risk After Radiation Therapy for Stage III Lung Cancer | Wang K | Int. J. Radiat. Oncol. Biol. Phys. | 2022 | 0 |
| 30 | Prognostic Value of Postinduction Chemotherapy Volumetric PET/CT Parameters for Stage IIIA or IIIB Non-Small Cell Lung Cancer Patients Receiving Definitive Chemoradiotherapy | Guberina M | J. Nucl. Med. | 2021 | 1 |
| 31 | Predictors of Pneumonitis After Conventionally Fractionated Radiotherapy for Locally Advanced Lung Cancer | Jolly S | Int. J. Radiat. Oncol. Biol. Phys. | 2021 | 3 |
| 32 | Cost-Effectiveness of Prophylactic Cranial Irradiation Versus MRI Surveillance for Extensive-Stage Small Cell Lung Cancer | Vargo JA | Int. J. Radiat. Oncol. Biol. Phys. | 2021 | 2 |
| 33 | Synchrotron Microbeam Radiation Therapy for the Treatment of Lung Carcinoma: A Preclinical Study | Djonov V | Int. J. Radiat. Oncol. Biol. Phys. | 2021 | 7 |
| 34 | Update of Incidence, Prevalence, Survival, and Initial Treatment in Patients With Non-Small Cell Lung Cancer in the US | Ganti AK | JAMA Oncol. | 2021 | 18 |
| 35 | Assessment of Prognostic Value of High- Sensitivity Cardiac Troponin T for Early Prediction of Chemoradiation Therapy-Induced Cardiotoxicity in Patients with Non-Small Cell Lung Cancer: A Secondary Analysis of a Prospective Randomized Trial | Liao ZX | Int. J. Radiat. Oncol. Biol. Phys. | 2021 | 2 |
| 36 | Concurrent Chemoradiation Therapy With or Without Nimotuzumab in Locally Advanced Squamous Cell Lung Cancer: A Phase 2 Randomized Trial | Liu H | Int. J. Radiat. Oncol. Biol. Phys. | 2021 | 1 |
| 37 | Randomized Phase III Trial of Prophylactic Cranial Irradiation With or Without Hippocampal Avoidance for Small-Cell Lung Cancer (PREMER): A GICOR-GOECP-SEOR Study | de Dios NR | J. Clin. Oncol. | 2021 | 22 |
| 38 | Stereotactic ablative radiotherapy for operable stage I non-small-cell lung cancer (revised STARS): long-term results of a single-arm, prospective trial with prespecified comparison to surgery | Chang JY | Lancet Oncol. | 2021 | 34 |
| 39 | Clinical Value of Upfront Cranial Radiation Therapy in Osimertinib-Treated Epidermal Growth Factor Receptor-Mutant Non-Small Cell Lung Cancer With Brain Metastases | Yu W | Int. J. Radiat. Oncol. Biol. Phys. | 2021 | 6 |
| 40 | Predicting Lymph Node Metastasis in Non-small Cell Lung Cancer Prospective External and Temporal Validation of the HAL and HOMER Models | Ost DE | Chest | 2021 | 4 |
| 41 | Moderately Hypofractionated Once-Daily Compared With Twice-Daily Thoracic Radiation Therapy Concurrently With Etoposide and Cisplatin in Limited-Stage Small Cell Lung Cancer: A Multicenter, Phase II, Randomized Trial | Liu H | Int. J. Radiat. Oncol. Biol. Phys. | 2021 | 10 |
| 42 | Ten-Year Experience in Implementing Single-Fraction Lung SBRT for Medically Inoperable Early-Stage Lung Cancer | Videtic GMM | Int. J. Radiat. Oncol. Biol. Phys. | 2021 | 5 |
| 43 | Accelerated Hypofractionated Image-Guided vs Conventional Radiotherapy for Patients With Stage II/III Non-Small Cell Lung Cancer and Poor Performance Status A Randomized Clinical Trial | Timmerman R | JAMA Oncol. | 2021 | 10 |
| 44 | Prophylactic Cranial Irradiation in Patients With High-Risk Metastatic Non-Small Cell Lung Cancer: Quality of Life and Neurocognitive Analysis of a Randomized Phase II Study | Arrieta O | Int. J. Radiat. Oncol. Biol. Phys. | 2021 | 2 |
| 45 | Stereotactic Body Radiation Therapy With a High Maximum Dose Improves Local Control, Cancer-Specific Death, and Overall Survival in Peripheral Early-Stage Non-Small Cell Lung Cancer | Takeda A | Int. J. Radiat. Oncol. Biol. Phys. | 2021 | 7 |
| 46 | Long-Term Outcomes From a Phase 2 Trial of Radiofrequency Ablation Combined With External Beam Radiation Therapy for Patients With Inoperable Non-Small Cell Lung Cancer | Farris MK | Int. J. Radiat. Oncol. Biol. Phys. | 2021 | 1 |
| 47 | Lung Cancer and Heart Disease Risks Associated With Low-Dose Pulmonary Radiotherapy to COVID-19 Patients With Different Background Risks | Shuryak I | Int. J. Radiat. Oncol. Biol. Phys. | 2021 | 6 |
| 48 | Addition of Metformin to Concurrent Chemoradiation in Patients With Locally Advanced Non-Small Cell Lung Cancer The NRG-LU001 Phase 2 Randomized Clinical Trial | Skinner H | JAMA Oncol. | 2021 | 13 |
| 49 | Metformin in Combination With Chemoradiotherapy in Locally Advanced Non-Small Cell Lung Cancer The OCOG-ALMERA Randomized Clinical Trial | Tsakiridis T | JAMA Oncol. | 2021 | 17 |
| 50 | Prophylactic Cranial Irradiation Reduces Brain Metastases and Improves Overall Survival in High-Risk Metastatic Non-Small Cell Lung Cancer Patients: A Randomized phase 2 Study (PRoT-BM trial) | Arrieta O | Int. J. Radiat. Oncol. Biol. Phys. | 2021 | 1 |
| 51 | Multiblock Discriminant Analysis of Integrative F-18-FDG-PET/CT Radiomics for Predicting Circulating Tumor Cells in Early-Stage Non-small Cell Lung Cancer Treated With Stereotactic Body Radiation Therapy | Lee SH | Int. J. Radiat. Oncol. Biol. Phys. | 2021 | 3 |
| 52 | Mean Heart Dose Is an Inadequate Surrogate for Left Anterior Descending Coronary Artery Dose and the Risk of Major Adverse Cardiac Events in Lung Cancer Radiation Therapy | Mak RH | Int. J. Radiat. Oncol. Biol. Phys. | 2021 | 9 |
| 53 | Proposed Quality Metrics for Lung Cancer Screening Programs A National Lung Cancer Roundtable Project | Mazzone PJ | Chest | 2021 | 5 |
| 54 | Effect of Postoperative Radiotherapy for Patients With pIIIA-N2 Non-Small Cell Lung Cancer After Complete Resection and Adjuvant Chemotherapy The Phase 3 PORT-C Randomized Clinical Trial | Wang LH | JAMA Oncol. | 2021 | 36 |
| 55 | Pembrolizumab Plus Concurrent Chemoradiation Therapy in Patients With Unresectable, Locally Advanced, Stage III Non-Small Cell Lung Cancer The Phase 2 KEYNOTE-799 Nonrandomized Trial | Jabbour SK | JAMA Oncol. | 2021 | 29 |
| 56 | The Plasma Levels and Polymorphisms of Vitronectin Predict Radiation Pneumonitis in Patients With Lung Cancer Receiving Thoracic Radiation Therapy | Cai XW | Int. J. Radiat. Oncol. Biol. Phys. | 2021 | 0 |
| 57 | Integrating Multiomics Information in Deep Learning Architectures for Joint Actuarial Outcome Prediction in Non-Small Cell Lung Cancer Patients After Radiation Therapy | Cui SN | Int. J. Radiat. Oncol. Biol. Phys. | 2021 | 6 |
| 58 | Neoadjuvant durvalumab with or without stereotactic body radiotherapy in patients with early-stage non-small-cell lung cancer: a single-centre, randomised phase 2 trial | Altorki NK | Lancet Oncol. | 2021 | 53 |
| 59 | GLUT1 Expression in Tumor-Associated Neutrophils Promotes Lung Cancer Growth and Resistance to Radiotherapy | Meylan E | Cancer Res. | 2021 | 24 |
| 60 | F-18-FLT PET/CT Adds Value to F-18-FDG PET/CT for Diagnosing Relapse After Definitive Radiotherapy in Patients with Lung Cancer: Results of a Prospective Clinical Trial | Christensen TN | J. Nucl. Med. | 2021 | 2 |
| 61 | Local Control After Stereotactic Body Radiation Therapy for Stage I Non-Small Cell Lung Cancer | Lee P | Int. J. Radiat. Oncol. Biol. Phys. | 2021 | 16 |
| 62 | Assessment of a Contralateral Esophagus-Sparing Technique in Locally Advanced Lung Cancer Treated With High-Dose Chemoradiation A Phase 1 Nonrandomized Clinical Trial | Willers H | JAMA Oncol. | 2021 | 9 |
| 63 | Institutional-Level Differences in Quality and Outcomes of Lung Cancer Resections in the United States | Osarogiagbon RU | Chest | 2021 | 4 |
| 64 | Treatment Monitoring of Immunotherapy and Targeted Therapy Using F-18-FET PET in Patients with Melanoma and Lung Cancer Brain Metastases: Initial Experiences | Galldiks N | J. Nucl. Med. | 2021 | 12 |
| 65 | Olaparib increases the therapeutic index of hemithoracic irradiation compared with hemithoracic irradiation alone in a mouse lung cancer model | Ryan AJ | Br. J. Cancer | 2021 | 0 |
| 66 | Effect of Second-generation vs Third-generation Chemotherapy Regimens With Thoracic Radiotherapy on Unresectable Stage III Non-Small-Cell Lung Cancer 10-Year Follow-up of a WJTOG0105 Phase 3 Randomized Clinical Trial | Zenke Y | JAMA Oncol. | 2021 | 2 |
| 67 | Isotoxic Intensity Modulated Radiation Therapy in Stage III Non-Small Cell Lung Cancer: A Feasibility Study | Faivre-Finn C | Int. J. Radiat. Oncol. Biol. Phys. | 2021 | 4 |
| 68 | Erlotinib Versus Etoposide/Cisplatin With Radiation Therapy in Unresectable Stage III Epidermal Growth Factor Receptor Mutation-Positive Non-Small Cell Lung Cancer: A Multicenter, Randomized, Open-Label, Phase 2 Trial | Yu JM | Int. J. Radiat. Oncol. Biol. Phys. | 2021 | 10 |
| 69 | Concurrent Chemo-Proton Therapy Using Adaptive Planning for Unresectable Stage 3 Non-Small Cell Lung Cancer: A Phase 2 Study | Iwata H | Int. J. Radiat. Oncol. Biol. Phys. | 2021 | 5 |
| 70 | Effects of Ultra-high doserate FLASH Irradiation on the Tumor Microenvironment in Lewis Lung Carcinoma: Role of Myosin Light Chain | Ahn GO | Int. J. Radiat. Oncol. Biol. Phys. | 2021 | 12 |
| 71 | Integration of Risk Survival Measures Estimated From Pre- and Posttreatment Computed Tomography Scans Improves Stratification of Patients With Early-Stage Non-small Cell Lung Cancer Treated With Stereotactic Body Radiation Therapy | Fan Y | Int. J. Radiat. Oncol. Biol. Phys. | 2021 | 1 |
| 72 | High-dose versus standard-dose twice-daily thoracic radiotherapy for patients with limited stage small-cell lung cancer: an open-label, randomised, phase 2 trial | Gronberg BH | Lancet Oncol. | 2021 | 32 |
| 73 | Early Changes in Physical Activity and Quality of Life With Thoracic Radiation Therapy in Breast Cancer, Lung Cancer, and Lymphoma | Ky B | Int. J. Radiat. Oncol. Biol. Phys. | 2021 | 0 |
| 74 | FAPI-74 PET/CT Using Either F-18-AlF or Cold-Kit Ga-68 Labeling: Biodistribution, Radiation Dosimetry, and Tumor Delineation in Lung Cancer Patients | Haberkorn U | J. Nucl. Med. | 2021 | 71 |
| 75 | Prospective Single-Arm Phase 1 and 2 Study: Ipilimumab and Nivolumab With Thoracic Radiation Therapy After Platinum Chemotherapy in Extensive-Stage Small Cell Lung Cancer | Perez BA | Int. J. Radiat. Oncol. Biol. Phys. | 2021 | 8 |
| 76 | Durvalumab, with or without tremelimumab, plus platinum-etoposide versus platinum-etoposide alone in first-line treatment of extensive-stage small-cell lung cancer (CASPIAN): updated results from a randomised, controlled, open-label, phase 3 trial | Paz-Ares L | Lancet Oncol. | 2021 | 112 |
| 77 | Association of Left Anterior Descending Coronary Artery Radiation Dose With Major Adverse Cardiac Events and Mortality in Patients With Non-Small Cell Lung Cancer | Mak RH | JAMA Oncol. | 2021 | 39 |
| 78 | Comparison of Different Methods for Delineation of F-18-FDG PET-Positive Tissue for Target Volume Definition in Radiotherapy of Patients with Non-Small Cell Lung Cancer | Nestle U | J. Nucl. Med. | 2020 | 0 |
| 79 | Hypofractionated Proton Therapy with Concurrent Chemotherapy for Locally Advanced Non-Small Cell Lung Cancer: A Phase 1 Trial from the University of Florida and Proton Collaborative Group | Hoppe BS | Int. J. Radiat. Oncol. Biol. Phys. | 2020 | 17 |
| 80 | Correlating Dose Variables with Local Tumor Control in Stereotactic Body Radiation Therapy for Early-Stage Non-Small Cell Lung Cancer: A Modeling Study on 1500 Individual Treatments | Klement RJ | Int. J. Radiat. Oncol. Biol. Phys. | 2020 | 18 |
| 81 | Is There a Role for Hypofractionated Thoracic Radiation Therapy in Limited-Stage Small Cell Lung Cancer? A Propensity Score Matched Analysis | Louie AV | Int. J. Radiat. Oncol. Biol. Phys. | 2020 | 10 |
| 82 | Central Airway Toxicity After High Dose Radiation: A Combined Analysis of Prospective Clinical Trials for Non-Small Cell Lung Cancer | Kong FM | Int. J. Radiat. Oncol. Biol. Phys. | 2020 | 4 |
| 83 | Cardiac Irradiation Predicts Activity Decline in Patients Receiving Concurrent Chemoradiation for Locally Advanced Lung Cancer | Ohri N | Int. J. Radiat. Oncol. Biol. Phys. | 2020 | 1 |
| 84 | CDK5 Activates Hippo Signaling to Confer Resistance to Radiation Therapy Via Upregulating TAZ in Lung Cancer | Liu L; Wu G; Xu SB | Int. J. Radiat. Oncol. Biol. Phys. | 2020 | 7 |
| 85 | Y Chromosome LncRNA Are Involved in Radiation Response of Male Non-Small Cell Lung Cancer Cells | Martinez I | Cancer Res. | 2020 | 10 |
| 86 | CB11, a novel purine-based PPAR gamma ligand, overcomes radio-resistance by regulating ATM signalling and EMT in human non-small-cell lung cancer cells | Hong SH | Br. J. Cancer | 2020 | 5 |
| 87 | A Pilot Study of Atezolizumab Plus Hypofractionated Image Guided Radiation Therapy for the Treatment of Advanced Non-Small Cell Lung Cancer | Ramnath N | Int. J. Radiat. Oncol. Biol. Phys. | 2020 | 6 |
| 88 | Effect of Low-Dose Radiation Therapy on Abscopal Responses to Hypofractionated Radiation Therapy and Anti-PD1 in Mice and Patients With Non-Small Cell Lung Cancer | Lu Y | Int. J. Radiat. Oncol. Biol. Phys. | 2020 | 19 |
| 89 | Programmed Death Receptor Ligand One Expression May Independently Predict Survival in Patients With Non-Small Cell Lung Carcinoma Brain Metastases Receiving Immunotherapy | Phillips JG | Int. J. Radiat. Oncol. Biol. Phys. | 2020 | 7 |
| 90 | Survival and Toxicity of Hypofractionated Intensity Modulated Radiation Therapy in 4 Gy Fractions for Unresectable Stage III Non-Small Cell Lung Cancer | Zhu XZ | Int. J. Radiat. Oncol. Biol. Phys. | 2020 | 7 |
| 91 | Evaluation of First-line Radiosurgery vs Whole-Brain Radiotherapy for Small Cell Lung Cancer Brain Metastases The FIRE-SCLC Cohort Study | Rusthoven CG | JAMA Oncol. | 2020 | 61 |
| 92 | SHP-2 and PD-L1 Inhibition Combined with Radiotherapy Enhances Systemic Antitumor Effects in an Anti-PD-1-Resistant Model of Non-Small Cell Lung Cancer | Welsh JW | Cancer Immunol. Res. | 2020 | 26 |
| 93 | Phase 1 Trial of Pembrolizumab Administered Concurrently With Chemoradiotherapy for Locally Advanced Non-Small Cell Lung Cancer A Nonrandomized Controlled Trial | Jabbour SK | JAMA Oncol. | 2020 | 46 |
| 94 | Pretreatment F-18-FDG PET/CT Radiomics Predict Local Recurrence in Patients Treated with Stereotactic Body Radiotherapy for Early-Stage Non-Small Cell Lung Cancer: A Multicentric Study | Dissaux G | J. Nucl. Med. | 2020 | 66 |
| 95 | Definitive Management of Presumed Synchronous Early Stage Non-Small Cell Lung Cancers: Outcomes and Utility of Stereotactic Ablative Radiation Therapy | Chang JY | Int. J. Radiat. Oncol. Biol. Phys. | 2020 | 3 |
| 96 | Impact of Early Prophylactic Cranial Irradiation With Hippocampal Avoidance on Neurocognitive Function in Patients With Limited Disease Small Cell Lung Cancer. A Multicenter Phase 2 Trial (SAKK 15/12) | Vees H | Int. J. Radiat. Oncol. Biol. Phys. | 2020 | 6 |
| 97 | Integrin alpha v beta 6 Positron Emission Tomography Imaging in Lung Cancer Patients Treated With Pulmonary Radiation Therapy | Saleem A | Int. J. Radiat. Oncol. Biol. Phys. | 2020 | 4 |
| 98 | Pembrolizumab for management of patients with NSCLC and brain metastases: long-term results and biomarker analysis from a non -randomised, open -label, phase 2 trial | Goldberg SB | Lancet Oncol. | 2020 | 170 |
| 99 | Pattern of Recurrence Analysis in Metastatic EGFR-Mutant NSCLC Treated with Osimertinib: Implications for Consolidative Stereotactic Body Radiation Therapy | Zhu ZF | Int. J. Radiat. Oncol. Biol. Phys. | 2020 | 16 |
| 100 | Dose to Highly Functional Ventilation Zones Improves Prediction of Radiation Pneumonitis for Proton and Photon Lung Cancer Radiation Therapy | Zou W | Int. J. Radiat. Oncol. Biol. Phys. | 2020 | 9 |
| 101 | To Biopsy or Not to Biopsy?: A Matched Cohort Analysis of Early -Stage Lung Cancer Treated with Stereotactic Radiation with or Without Histologic Confirmation | Dautruche A | Int. J. Radiat. Oncol. Biol. Phys. | 2020 | 3 |
| 102 | A Comparison of Radiation Techniques in Patients Treated With Concurrent Chemoradiation for Stage III Non-Small Cell Lung Cancer | Swaminath A | Int. J. Radiat. Oncol. Biol. Phys. | 2020 | 13 |
| 103 | Evaluating Positron Emission Tomography-Based Functional Imaging Changes in the Heart After Chemo-Radiation for Patients With Lung Cancer | Vinogradskiy Y | Int. J. Radiat. Oncol. Biol. Phys. | 2020 | 7 |
| 104 | Imaging-based target volume reduction in chemoradiotherapy for locally advanced non-small-cell lung cancer (PET-Plan): a multicentre, open-label, randomised, controlled trial | Nestle U | Lancet Oncol. | 2020 | 54 |
| 105 | Long-Term Results from the IDEAL-CRT Phase 1/2 Trial of Isotoxically Dose-Escalated Radiation Therapy and Concurrent Chemotherapy for Stage II/III Non-small Cell Lung Cancer | Landau DB | Int. J. Radiat. Oncol. Biol. Phys. | 2020 | 10 |
| 106 | Long-Term Results of NRG Oncology RTOG 0617: Standard- Versus High-Dose Chemoradiotherapy With or Without Cetuximab for Unresectable Stage III Non-Small-Cell Lung Cancer | Bradley JD | J. Clin. Oncol. | 2020 | 170 |
| 107 | Immune and Circulating Tumor DNA Profiling After Radiation Treatment for Oligometastatic Non-Small Cell Lung Cancer: Translational Correlatives from a Mature Randomized Phase II Trial | Zhang JJ | Int. J. Radiat. Oncol. Biol. Phys. | 2020 | 16 |
| 108 | Early Tumor and Nodal Response in Patients with Locally Advanced Non-Small Cell Lung Carcinoma Predict for Oncologic Outcomes in Patients Treated with Concurrent Proton Therapy and Chemotherapy | Berman AT | Int. J. Radiat. Oncol. Biol. Phys. | 2020 | 2 |
| 109 | Proton Beam Therapy for Histologically or Clinically Diagnosed Stage I Non-Small Cell Lung Cancer (NSCLC): The First Nationwide Retrospective Study in Japan | Sakurai H | Int. J. Radiat. Oncol. Biol. Phys. | 2020 | 8 |
| 110 | Minocycline Reduces Chemoradiation-Related Symptom Burden in Patients with Non-Small Cell Lung Cancer: A Phase 2 Randomized Trial | Wang XS | Int. J. Radiat. Oncol. Biol. Phys. | 2020 | 5 |
