# Supplementary material for: Research landscape and trends of lung cancer radiotherapy: A bibliometric analysis
Source: Front Oncol. 2022 Nov 10;12:1066557. doi: 10.3389/fonc.2022.1066557 (PMC9685815; doi:10.3389/fonc.2022.1066557)
Supplement: Supplementary Table S4 — The corresponding authors with at least two top-papers in lung cancer radiotherapy from 2000 to 2022. [file Table_4.docx]

| **TABLE S4** \| The corresponding authors with at least two top-papers in lung cancer radiotherapy from 2000 to 2022. | | | | | |
| --- | --- | --- | --- | --- | --- |
| **Corresponding author** | **Paper number** | **Total citation** | **Average citations per paper** | **Top-paper number** | **Representative work^a^** |
| Timmerman R | 8 | 3767 | 470.88 | 3 | Stereotactic Body Radiation Therapy for Inoperable Early Stage Lung Cancer |
| Onishi H | 8 | 2029 | 253.63 | 3 | Hypofractionated stereotactic radiotherapy (HypoFXSRT) for stage I non-small cell lung cancer: Updated results of 257 patients in a Japanese multi-institutional study |
| Lagerwaard FJ | 16 | 2061 | 128.81 | 3 | Outcomes of risk-adapted fractionated stereotactic radiotherapy for stage I non-small-cell lung cancer |
| vanMeerbeeck JP | 5 | 1174 | 234.80 | 2 | Small-cell lung cancer |
| Vokes EE | 11 | 762 | 69.27 | 2 | Induction chemotherapy followed by chemoradiotherapy compared with chemoradiotherapy alone for regionally advanced unresectable stage III non-small-cell lung cancer: Cancer and Leukemia Group B |
| Rosen LS | 2 | 1004 | 502.00 | 2 | Zoledronic acid versus placebo in the treatment of skeletal metastases in patients with lung cancer and other solid tumors: A phase III, double-blind, randomized trial - The zoledronic acid lung cancer and other solid tumors study group |
| Nagata Y | 4 | 836 | 209.00 | 2 | Clinical outcomes of a phase I/II study of 48 Gy of stereotactic body radiotherapy in 4 fractions for primary lung cancer using a stereotactic body frame |
| Gomez DR | 23 | 1588 | 69.04 | 2 | Local consolidative therapy versus maintenance therapy or observation for patients with oligometastatic non-small-cell lung cancer without progression after first-line systemic therapy: a multicentre, randomised, controlled, phase 2 study |
| Douillard JY | 2 | 1409 | 704.50 | 2 | Adjuvant vinorelbine plus cisplatin versus observation in patients with completely resected stage IB-IIIA non-small-cell lung cancer (Adjuvant Navelbine International Trialist Association [ANITA]): a randomised controlled trial |
| Albain KS | 2 | 1248 | 624.00 | 2 | Radiotherapy plus chemotherapy with or without surgical resection for stage III non-small-cell lung cancer: a phase III randomised controlled trial |
| Bradley J | 5 | 934 | 186.80 | 2 | Impact of FDG-PET on radiation therapy volume delineation in non-small-cell lung cancer |
| Antonia SJ | 2 | 3468 | 1734.00 | 2 | Durvalumab after Chemoradiotherapy in Stage III Non-Small-Cell Lung Cancer |
| ^a^The most cited paper in lung cancer radiotherapy of the corresponding author. | | | | | |
