# Supplementary figures and images for: Research landscape and trends of lung cancer radiotherapy: A bibliometric analysis
Source: Front Oncol. 2022 Nov 10;12:1066557. doi: 10.3389/fonc.2022.1066557 (PMC9685815; doi:10.3389/fonc.2022.1066557)

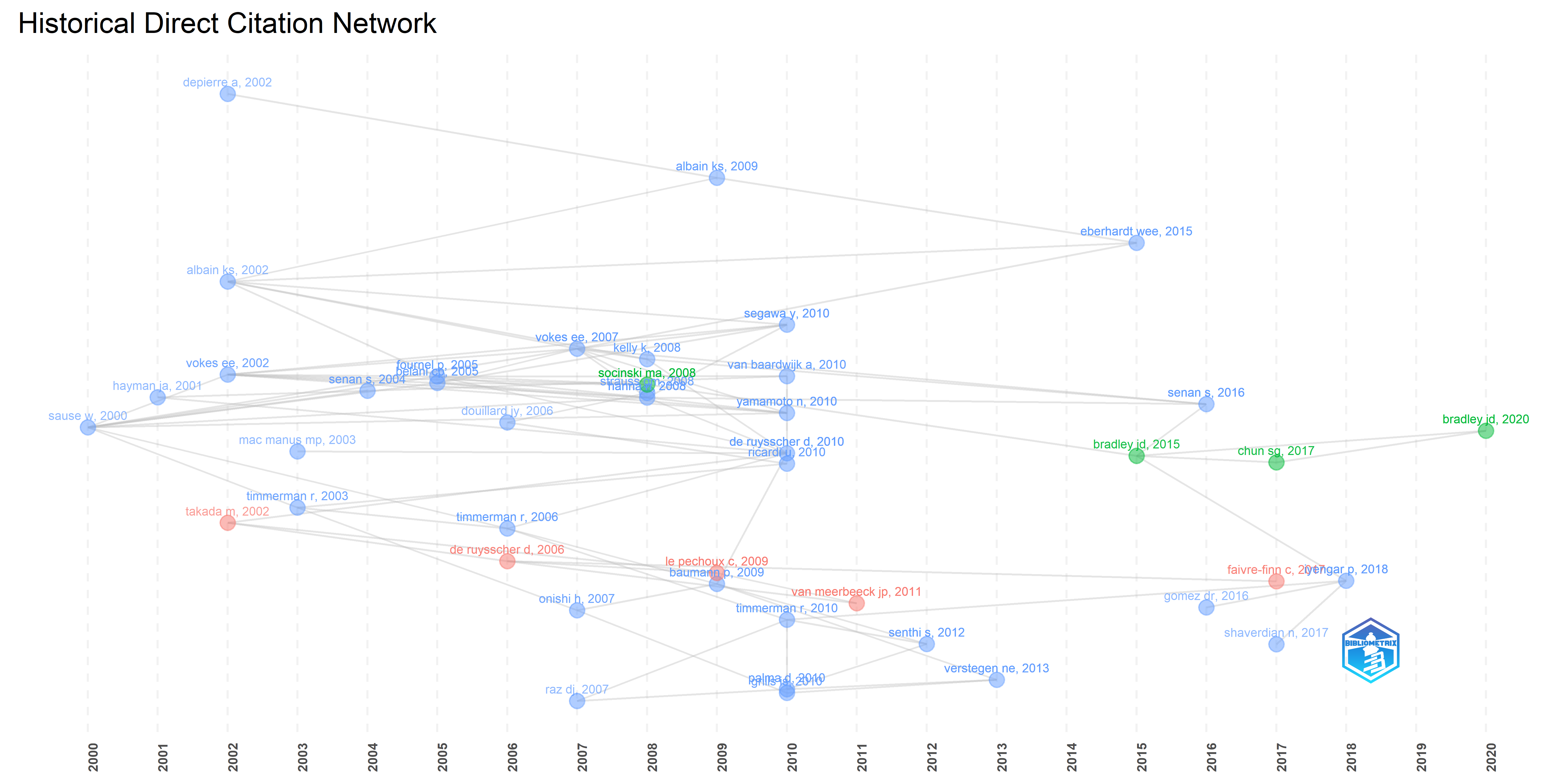

Supplement: Supplementary Figure 1 — Historical direct citation network among the publications. [file Image_1.png]

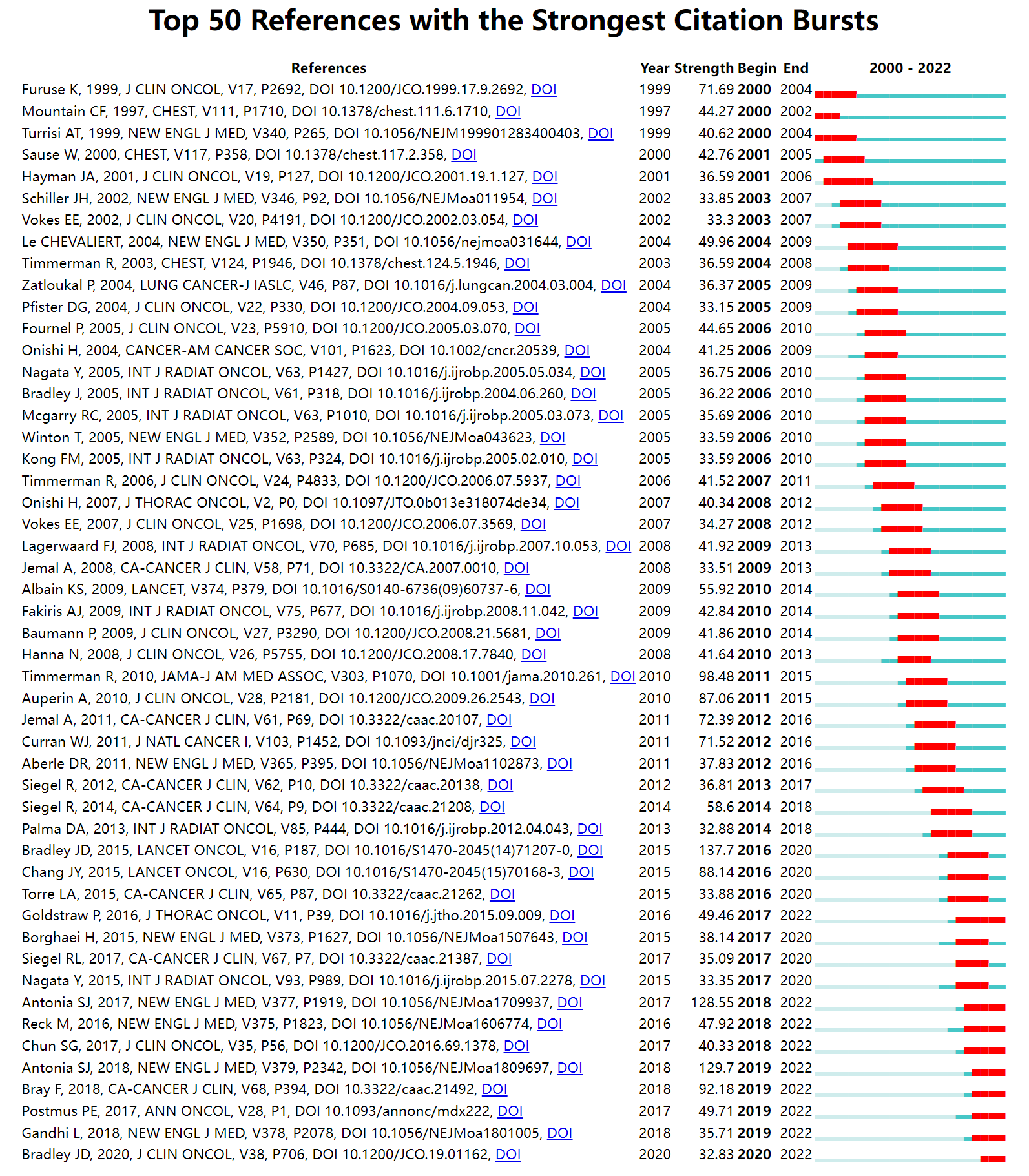

Supplement: Supplementary Figure 2 — Top 50 references with the strongest citation bursts on lung cancer radiotherapy. [file Image_2.png]

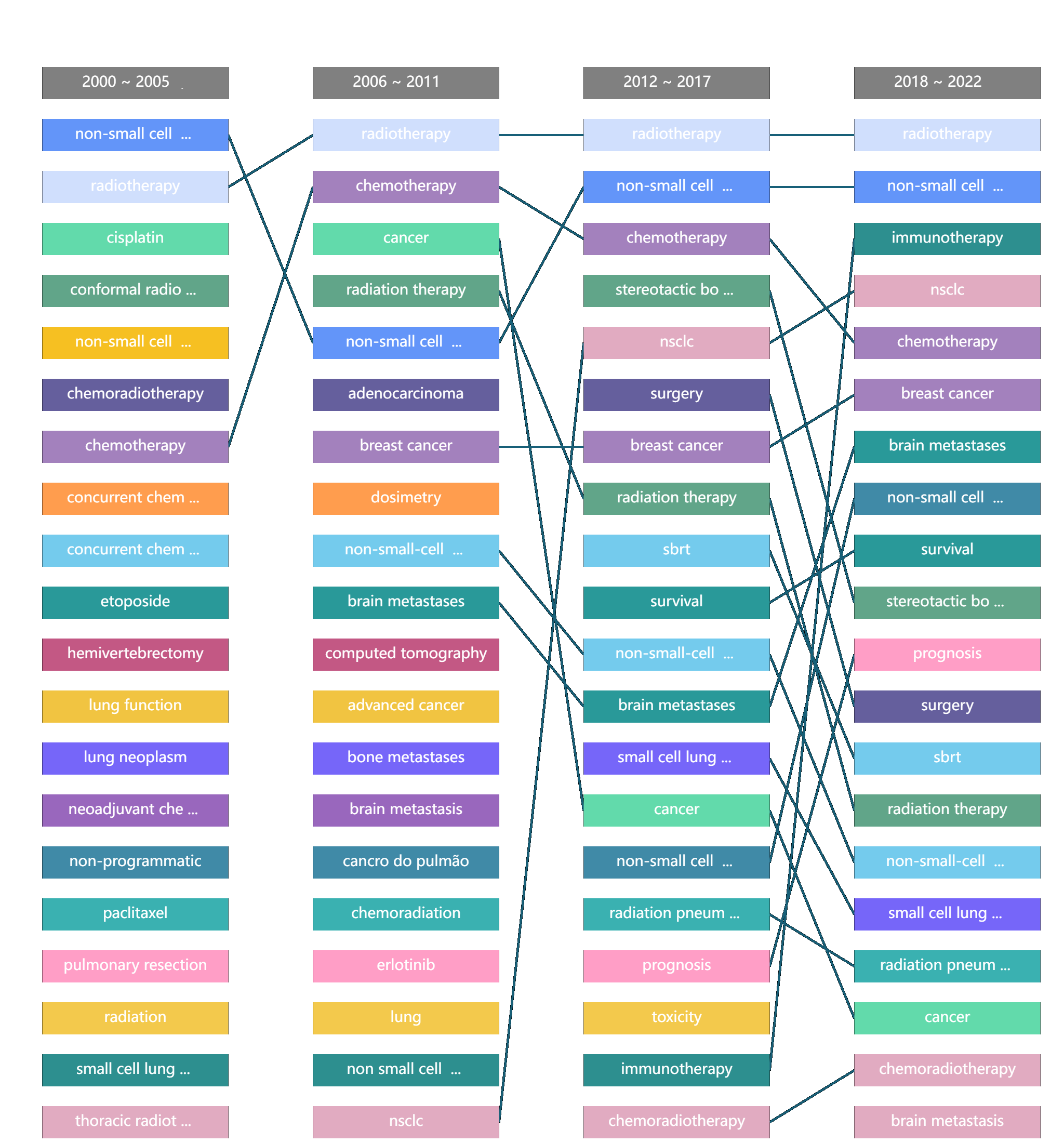

Supplement: Supplementary Figure 4 — The trends of the keywords rank of lung cancer radiotherapy. [file Image_4.png]

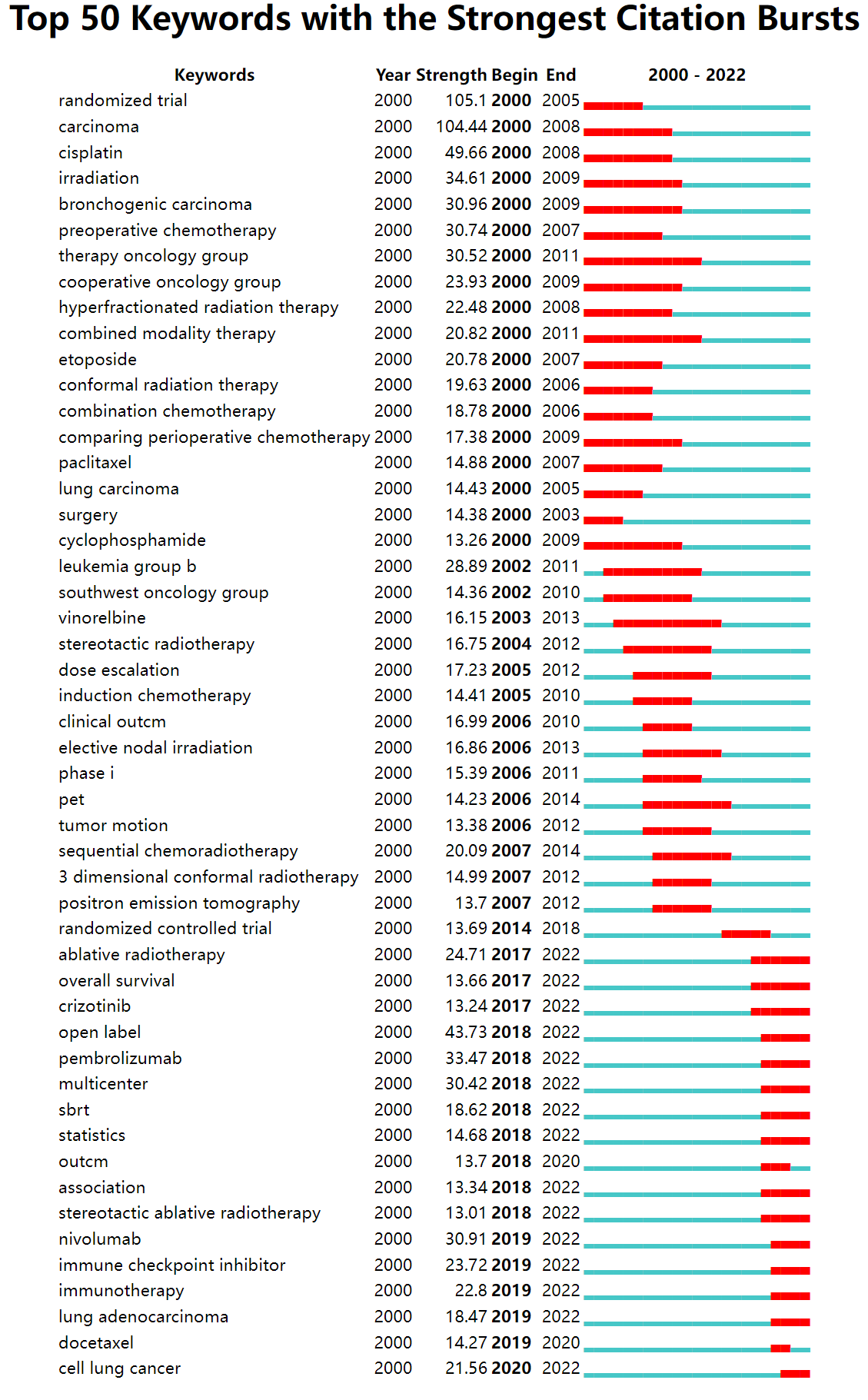

Supplement: Supplementary Figure 5 — Top 50 keywords with the strongest citation bursts on lung cancer radiotherapy. [file Image_5.png]
